# Deep learning methods hold promise for light fluence compensation in three-dimensional optoacoustic imaging

**DOI:** 10.1117/1.JBO.27.10.106004

**Published:** 2022-10-08

**Authors:** Arumugaraj Madasamy, Vipul Gujrati, Vasilis Ntziachristos, Jaya Prakash

**Affiliations:** a Indian Institute of Science, Department of Instrumentation and Applied Physics, Bengaluru, Karnataka, India; b Institute of Biological and Medical Imaging, Helmholtz Zentrum München (GmbH), Neuherberg, Germany; c Technical University of Munich, School of Medicine, Chair of Biological Imaging, Munich, Germany; d Technical University of Munich, Munich Institute of Robotics and Machine Intelligence (MIRMI), Munich, Germany

**Keywords:** optoacoustic imaging, fluence correction, image deconvolution, image reconstruction, deep learning

## Abstract

**Significance:** Quantitative optoacoustic imaging (QOAI) continues to be a challenge due to the influence of nonlinear optical fluence distribution, which distorts the optoacoustic image representation. Nonlinear optical fluence correction in OA imaging is highly ill-posed, leading to the inaccurate recovery of optical absorption maps. This work aims to recover the optical absorption maps using deep learning (DL) approach by correcting for the fluence effect.

**Aim:** Different DL models were compared and investigated to enable optical absorption coefficient recovery at a particular wavelength in a nonhomogeneous foreground and background medium.

**Approach:** Data-driven models were trained with two-dimensional (2D) Blood vessel and three-dimensional (3D) numerical breast phantom with highly heterogeneous/realistic structures to correct for the nonlinear optical fluence distribution. The trained DL models such as U-Net, Fully Dense (FD) U-Net, Y-Net, FD Y-Net, Deep residual U-Net (Deep ResU-Net), and generative adversarial network (GAN) were tested to evaluate the performance of optical absorption coefficient recovery (or fluence compensation) with *in-silico* and *in-vivo* datasets.

**Results:** The results indicated that FD U-Net-based deconvolution improves by about 10% over reconstructed optoacoustic images in terms of peak-signal-to-noise ratio. Further, it was observed that DL models can indeed highlight deep-seated structures with higher contrast due to fluence compensation. Importantly, the DL models were found to be about 17 times faster than solving diffusion equation for fluence correction.

**Conclusions:** The DL methods were able to compensate for nonlinear optical fluence distribution more effectively and improve the optoacoustic image quality.

## 1 Introduction

Optoacoustic imaging (OAI) relies on the optoacoustic effect.^1^ OAI provides optical contrast at acoustic resolution.^2^^,^^3^ Longitudinal noninvasive volumetric imaging of intrinsic bio-molecules such as oxyhemoglobin, deoxyhemoglobin, lipids, water, and extrinsic molecules are very important for treatment monitoring and therapy response.^4^^,^^5^ OAI involves illuminating the sample with a tunable nano-second pulsed laser, this illuminated light is absorbed and scattered by the biological tissue. The absorbed light generates a small rise in temperature in the order of milli-Kelvin, which then generates broadband acoustic waves having frequencies in the range of kHz to a few tens of MHz. The broadband acoustic waves are then detected using an ultrasound transducer (either single element or arrays) in a tomographic fashion, the recorded acoustic waves are then used to reconstruct the initial pressure rise distribution by solving an acoustic inverse problem. Note that the wavelength of the illuminated light can be varied to obtain multispectral OAI data, which has the ability to resolve different intrinsic biological chromophores such as oxyhemoglobin, deoxyhemoglobin, lipids, and water.^6^^–^^12^ The reconstructed initial pressure rise signal is a product of optical fluence, the optical absorption coefficient, and the Grüneisen parameter.^13^

OAI requires highly sensitive ultrasound detectors for collecting weak OA signals generated from deeper biological tissues. Also, fluence compensation over different wavelengths to unmix different chromophores is challenging due to the nonlinear variation of fluence distribution.^14^ Many OAI studies focus on obtaining the initial pressure rise distribution from the acquired tomographic acoustic measurements wherein the optical fluence is assumed to be constant.^15^^,^^16^ This assumption is not valid for optoacoustic tomographic imaging setups and optical fluence influences the reconstructed optoacoustic images. The optical fluence decreases exponentially as a function of imaging depth due to absorption and scattering events that occur inside the medium under investigation.^17^ Further the optical fluence distribution changes with wavelength since optical absorption and scattering are nonlinear function of wavelength of light in the biological tissue.^18^ This depth and wavelength-dependent optical fluence variations should be considered while recovering optical absorption coefficient or resolving the chromophore distribution. Hence accurate recovery of optical absorption coefficient remains challenging in OAI.^19^^,^^20^ The nonlinear variation of the fluence^14^^,^^16^ will affect the generation and propagation of the OA wave.

The optical absorption coefficient distribution could be estimated from the initial pressure rise distribution by correcting/compensating for the local optical fluence. Fluence compensation^16^^,^^20^^–^^28^ to estimate absorption coefficient ($\mu_{\mathrm{aest}}$) can be accomplished by dividing the initial pressure rise distribution ($P_{o}$) with the optical fluence (ϕ), which can be expressed as (assuming the Grüneisen parameter to be unity)

$$\mu_{\mathrm{aest}}=P_{o}/\phi .$$ (1)

Note that the fluence correction method needs precise estimation of optical fluence map. Many different light transport models exist for estimating the optical fluence map when the optical properties, i.e., optical absorption and optical scattering are known. The light transport model can be performed using Monte Carlo,^29^ NIRFAST,^30^^–^^32^ or COMSOL^33^ toolboxes. Reference 34 performed both optical and acoustical compensation for OA tomography by assuming optical properties to be known. However in reality the optical properties are unknown, therefore fluence estimation becomes difficult during OA imaging. Alternatively, a rough estimate of optical fluence distribution can be obtained from different imaging modalities like diffuse optical tomography, and then the obtained fluence can be used to estimate the optical absorption coefficient distribution.^35^

Deep learning (DL) models have played a vital role in various medical imaging applications^36^^,^^37^ specifically in the context of denoising, super-resolution, and segmentation.^38^^–^^41^ A convolutional neural network (CNN) has the potential to learn features and embeddings from imaging data. The DL models were used to get the target output either via preprocessing or post-processing operation. DL methods were also employed for data visualization, reconstruction, and improve/enhance OAI.^40^^–^^52^ Recently, a few DL methods were proposed for fluence compensation in OAI,^42^^,^^44^^–^^46^^,^^53^^,^^54^ but those studies considered simple phantoms such as point/dot, discs, line phantoms, and circle phantom.^44^^,^^46^^,^^53^ Very less emphasis has been placed on evaluating the performance of DL models for fluence compensation on *ex-vivo*^54^ or *in-vivo*^44^^,^^54^ data.

In this paper, we explored different DL approaches to compensate for light fluence distribution from the reconstructed initial pressure rise and evaluated the performance of different DL methods with two-dimensional (2D) blood vessel (BV) network, three-dimensional (3D) numerical breast phantom, and 2D *in-vivo* dataset. In our study, light fluence compensation was performed and evaluated using different DL networks, wherein the input OA images were reconstructed by a conventional method like backprojection. The reconstructed images were affected by different factors such as optical fluence, noise, and blur, these factors are known to reduce the image quality. The degraded images/volumes are then improved by a DL model, which is expected to correct the light fluence, noise, and other artifacts. To the best of our knowledge, this was the first method that considered more realistic vasculature distribution (not having binary representation) in 2D and 3D numerical phantoms for accurate recovery of optical absorption coefficient distribution using the DL platform. Further *in-vivo* evaluation was also performed using different DL methods such as U-Net,^55^ FD U-Net,^47^ Y-Net,^56^ FD Y-Net,^56^ Deep ResU-Net,^57^ and GAN.^58^

The main contributions of this paper are as follows: (1) Well-characterized and complex 2D blood vasculature phantom having realistic foreground and background distribution are considered for the simulation study. Appropriate optical and acoustical properties are assigned to the phantom. (2) The performance of the different DL models to compensate for the optical fluence in the reconstructed images by varying wavelength and noise level were analyzed. In addition, we analyzed the generalization of DL models with *in-vivo* data. (3) The computational times and accuracy of the different DL models were studied using a 3D numerical breast phantom, which could be applied for 3D whole breast OAI without compressing the breast tissue. (4) Lastly, the parameters such as layers, batch size, and convergence were optimized for the DL-based fluence correction model.

## 2 Materials and Methods

### 2.1 Optoacoustic Imaging Process

The OA imaging relies on a “light in and sound out” approach, i.e., the OA effect. OAI is a combination of three processes namely, (1) optical process: The propagation of light and its interaction with the tissue (optical forward problem), (2) OA effect: Thermalization of the absorbed optical energy and generation of pressure waves in the tissue, and (3) acoustic process: The propagation of ultrasound (pressure) waves through the tissue and its detection using ultrasound transducers (acoustic forward problem). The detected acoustic signals are then converted into an OA image, i.e., the initial pressure rise distribution using a well-known image reconstruction algorithm, this procedure is often called the acoustic inverse problem. The optical inverse problem in OAI involves recovering the optical absorption coefficient from the optical absorbed energy distribution.

### 2.2 Realistic Blood Vessel Extraction and Numerical Breast Phantom

**Fig. 1:**
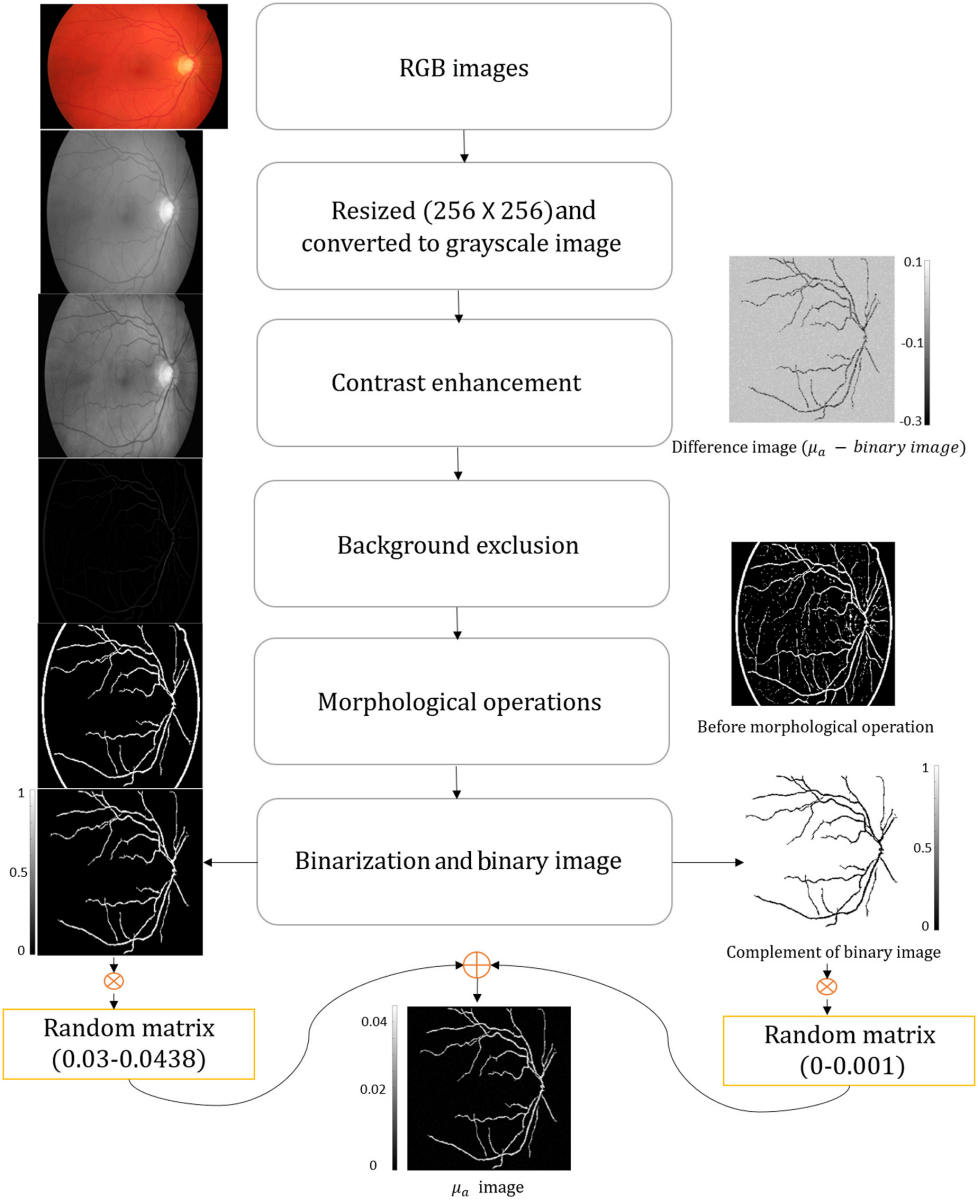
Image processing steps to generate realistic vasculature phantom for OA imaging from fundus data.

The BV extraction process^59^^,^^60^ from a fundus image is shown in Fig. 1. The extraction process starts by resizing the RGB image to the required size (256×256). The resized image is then converted into a gray-scale image using the rgb2gray function in MATLAB. Contrast-limited adaptive histogram equalization (CLAHE) algorithm is then used for enhancing the BV in the gray-scale image. Image smoothing was then performed using an average filter to improve the image quality. Isodata thresholding is applied on the difference image (obtained by subtracting the gray-scale vasculature network and enhanced image after image smoothing) to automatically find a threshold value for a given gray-scale image. After finding the threshold value, the im2bw function in MATLAB is used to generate a binary image. Bwareaopen operation has been applied to remove the small pixels or objects from the binary images. Isolated pixels from the binary image are further removed by the morphological operation, using the bwmorph(binaryImage,’clean’) function in MATLAB to retain only the connected vasculature network.

After all the above image processing steps, we extracted the vasculature from the fundus image dataset, which mimics a typically observed blood vasculature. We have generated two different random matrices. The random matrix is filled with random numbers and the range of these random numbers is controlled to be within physiologically relevant absorption levels. The random matrix corresponding to the foreground is estimated to be $A_{\text{fore}}=\mu_{{\mathrm{af}}_{\min}}+(\mu_{{\mathrm{af}}_{\max}}-\mu_{{\mathrm{af}}_{\min}})*\mathrm{rand}[\text{size}(A_{\text{fore}})]$; this random matrix is multiplied with the binary image, i.e., $X_{\text{fore}}=A_{\text{fore}}.*B$; where B is the original binary image. Next, a random matrix corresponding to the background is estimated to be $A_{\text{back}}=\mu_{{\mathrm{ab}}_{\min}}+(\mu_{{\mathrm{ab}}_{\max}}-\mu_{{\mathrm{ab}}_{\min}})*\mathrm{rand}[\text{size}(A_{\text{back}})]$; this random matrix is multiplied with the complement of the original binary image, i.e., $X_{\text{back}}=A_{\text{back}}.*\overset{¯}{B}$; where $\bar{B}$ is the complement of the original binary image. The resultant realistic numerical phantom was obtained as $X=X_{\text{back}}+X_{\text{fore}}$. The maximum and minimum absorption value for the foreground was set as $\mu_{{\mathrm{af}}_{\min}}=0.03$ and $\mu_{{\mathrm{af}}_{\max}}=0.0438\;{\mathrm{cm}}^{-1}$, whereas the maximum and minimum absorption value for the background was set as $\mu_{{\mathrm{ab}}_{\min}}=0$ and $\mu_{{\mathrm{ab}}_{\max}}=0.001\;{\mathrm{cm}}^{-1}$.

The imfill morphological operation was performed on 3D numerical breast phantom to fill image regions and holes. Further, the different layers in the breast phantom were assigned realistic optical absorption values corresponding to different tissue types in the numerical phantom.

### 2.3 Optical Forward Problem

The optical forward model in OA imaging describes how the light is transported through tissue or turbid medium. The light transport model can be mathematically expressed by radiative transfer equation (RTE) or diffusion equation (DE).^17^ When the light interacts with any tissue medium it exhibits two important phenomena namely, (1) optical absorption: optical light energy is absorbed by different types of chromophores that are present in the tissue, (2) scattering: light is redirected to different direction due to the presence of subcellular structures and small cells. In this paper, DE-based light propagation was used.

#### 2.3.1 Diffusion equation

The RTE equation is an integrodifferential equation that has many independent parameters.^17^ Obtaining an analytical solution for RTE is difficult because RTE has six independent parameters, hence RTE is often approximated to DE. The DE is a partial differential equation and can be solved by using the finite-element method (FEM). The continuous wave DE can be expressed as^61^

$$-\nabla \cdot [D(r)\nabla \phi (r)]+\mu_{a}(r)\phi (r)=q(r),$$ (2)

where ϕ(r) is the light fluence at the position r, $D(r)=\frac{1}{3(\mu_{a}+{\mu_{s}}^{\prime })}$ is the diffusion coefficient at position r, $\mu_{a}$ and $\mu_{s^{\prime }}$ are the optical absorption and reduced scattering coefficients, respectively, and q(r) is the light source. An estimate for light fluence can be obtained if the optical properties, i.e., D(r), $\mu_{a}(r)$, and source term, i.e., q(r) is known by solving the DE. In this work the DE is solved using the NIRFAST software,^30^^–^^32^ which uses FEM. A Robin-type boundary condition was used while solving the DE. For a known optical absorption coefficient, diffusion coefficient, and source term, we can compute the optical fluence distribution inside the tissue medium by solving Eq. (2) using NIRFAST toolbox.

### 2.4 Optical Simulation Geometry and Optical Properties in Tissue

In this work, a 2D square imaging region of 25 mm is considered, further, 10 sources were placed at the boundary (i.e., on the top of the square). The sources were illuminating along the y-direction. The total number of nodes and linear triangular elements in the grids are around 66,166 and 131,386, respectively. The optical properties of 2D model were set as 0.03 to $0.0438\;{\mathrm{cm}}^{-1}$ (foreground) and 0 to $0.001\;{\mathrm{cm}}^{-1}$ (background) optical absorption coefficients, optical scattering is kept constant in the imaging region as $1\;{\mathrm{cm}}^{-1}$, and refractive index is kept as 1.33.^62^

For the case of breast phantom, we considered a 3D cubic imaging region of 25 mm with ten sources placed at the boundary of the phantom. The source is illuminating in all directions to generate uniform illumination. The total number of nodes and linear tetrahedral elements in the volume is around 366,808 and 2,205,754, respectively. The optical absorption coefficient of 3D imaging model was set to be $0.016\;{\mathrm{mm}}^{-1}$ (for fibro-glandular region), $0.024\;{\mathrm{mm}}^{-1}$ (for fat region), $0.032\;{\mathrm{mm}}^{-1}$ (for skin region), $0.04\;{\mathrm{mm}}^{-1}$ (for vessels), and $0\;{\mathrm{mm}}^{-1}$ (for background).^63^ The remaining optical properties were considered to be the same as the 2D model. The optical simulation model was performed using NIRFAST light propagation model on a system running Ubuntu 20.04 LTS (Intel i9-9900K, 3.60 GHz, 64 GB RAM) in MATLAB 2021a.

#### 2.4.1 Absorbed optical energy computation

**Fig. 2:**
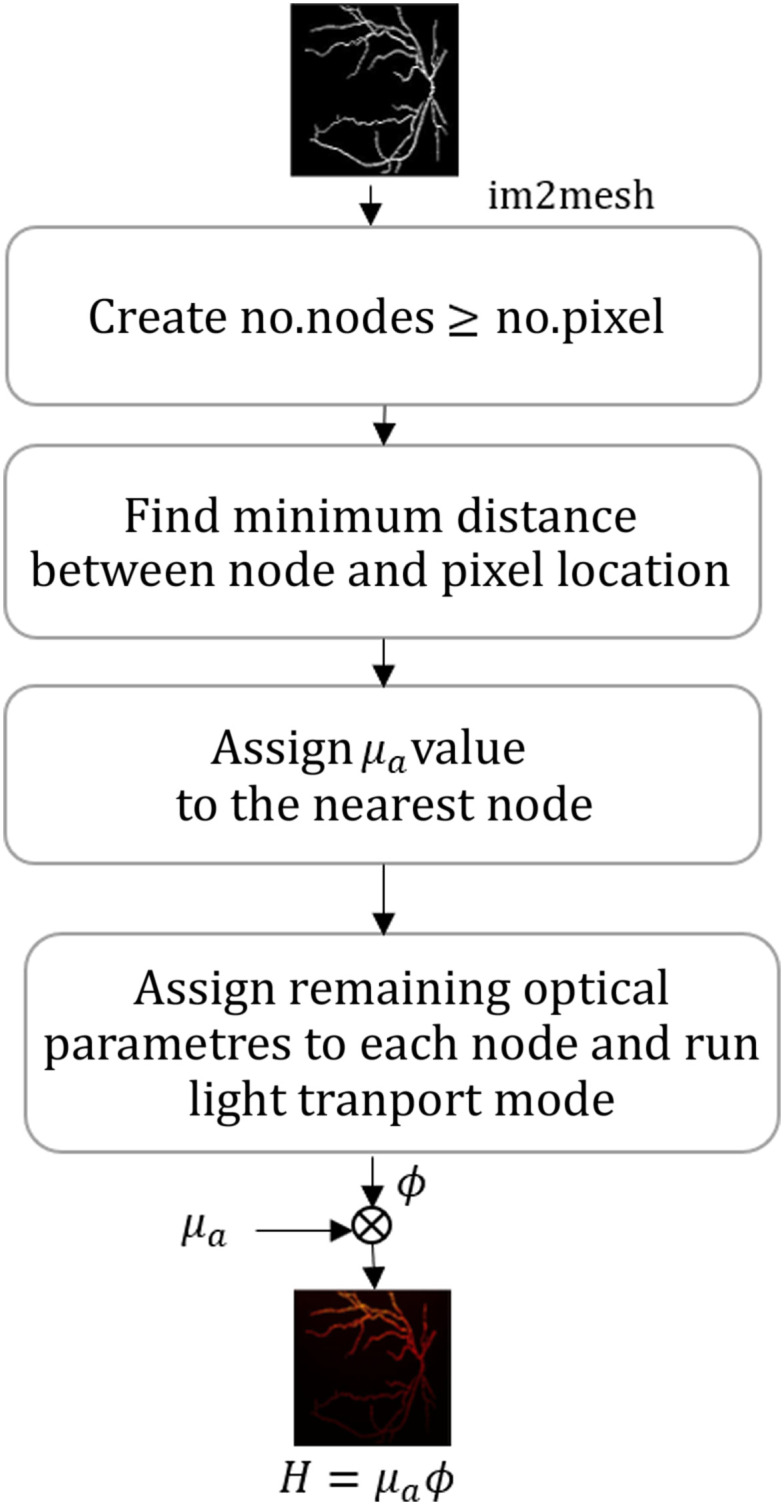
Flow chart indicating the conversion from mesh to image and estimation of absorbed optical energy distribution.

Note that the absorption coefficient is considered as an image, and the computed fluence is represented using nodes as a mesh. To estimate fluence, we need to convert the absorption image to a mesh format and assign relevant fields for enabling light propagation, which is performed as shown in Fig. 2. The binary absorption coefficient image is given as the input to im2mesh function in MATLAB, this function can generate a mesh having small triangular elements mapping the physical locations onto the image. In this workflow, we have generated the number of nodes in the mesh to be greater than or equal to the number of pixels in the image, as we did not want to miss any image pixel while assigning the optical properties to the corresponding mesh nodes. Once we have the image pixel location and mesh node location using im2mesh function. We then find the mesh node, which is nearer to the image pixel and assign the corresponding absorption value ($\mu_{a}$) in the image pixel value to the nearest node; this generates the mesh populated with relevant $\mu_{a}$ field. Next other optical properties are assigned to the mesh structure. Once the mesh structure is generated and appropriate fields are assigned, we run the forward model using NIRFAST, resulting in the optical fluence map in mesh format. This fluence map is then element-wise multiplied with $\mu_{a}$ (along the mesh), which gives the absorbed optical energy density as mesh image (corresponding to different node positions).

The absorbed optical energy distribution will be in a mesh format, which can be converted back into an image format by finding the minimum distance between the mesh node and pixel location. Once we have these distances, appropriate values can be assigned from the mesh to the image based on the shortest distance similar to nearest neighborhood approach. The computed absorbed optical energy distribution $H=\mu_{a}\times \Phi$ is also called the initial pressure rise distribution (by assuming Γ=1).

### 2.5 Acoustic Forward Problem

In this study, we assumed the medium to be acoustically homogeneous and lossless, further since nano-second pulses are used thermal and stress confinement conditions are satisfied. Under these conditions, the generated acoustic wave propagates through the tissue, which can be mathematically expressed as^64^

$$\nabla^{2}P(r,t)-\frac{1}{v^{2}}\frac{\partial^{2}P(r,t)}{\partial t^{2}}=-\frac{\beta }{C_{p}}H(r,t),$$ (3)

where v, β, $C_{p}$, P(r,t), and H(r,t) represent the speed of sound, thermal expansion coefficient, specific heat capacity at constant pressure, acoustic pressure, heat energy per unit volume, and per unit time deposited at a location r, respectively. The above equation can be rewritten as an initial value problem, which can be expressed as^64^

$$\nabla^{2}P(r,t)-\frac{1}{v^{2}}\frac{\partial^{2}P(r,t)}{\partial t^{2}}=0,$$ (4)

where the initial conditions are given as^64^

$$P(r,t){|}_{t=0}=P_{o}(r)=\Gamma (r)H(r)\;{\frac{\partial P(r,t)}{\partial t}|}_{t=0}=0.$$ (5)

If we assume the medium to be infinite by considering a delta heating source, then we can get the pseudospectral solution to Eq. (4). The propagated OA waves are then recorded by the transducer over a time interval 0 to T outside the tissue using k-wave software.^65^

#### 2.5.1 Acoustic simulation geometry and acoustic properties

The acoustic forward model is used for recording the acoustic sinogram data at the transducer (sensor) position. Herein, we have considered a 2D computational grid having a size of 801×801 grid points (80.1  mm×80.1  mm) with a distance of 0.05 mm/grid point for generating the sinogram data. The sensors were placed on a circle of 19-mm radius, and 256 detectors were placed equidistantly on this circle. The detector had a center frequency of 5 MHz and 90% fractional bandwidth and the speed of sound was assumed to be 1540  m/s. A perfectly matched layer (PML) was used to attenuate the pressure signal thereby avoiding any reflection of pressure signal into the reconstruction grid. The PML layer was placed over the edge of the domain and occupied 20 grid points. The time step for data collection was 50 ns, with a total of 2048 steps (256×2048=524,288). The acoustic inverse model is used for image reconstruction having a grid size of 401×401 (40.05  mm×40.05  mm) with a distance of 0.1  mm/grid point to avoid inverse crime.

While solving the problem in 3D, we considered a computational grid having a size of 131×131×131 grid points (25  mm×25  mm×25  mm) with a distance of 0.19  mm/grid point while generating the sinogram data. For 3D data acquisition, planar detectors were placed at the edge of the computational grid. The detector had a center frequency of 5 MHz and 90% fractional bandwidth and the speed of sound was set to be 1540  m/s. Similar to 2D case a PML with 20 grid points was used. The time step for data collection was 50 ns, with a total of 2391 steps (n=4096×2391=9,793,536).

#### 2.5.2 Acoustic inverse problem

The first step is to obtain the OA images, i.e., the initial pressure distribution, which can be mathematically expressed as^7^

$$P_{o}(r,\lambda )=\Gamma (r)H(r,\lambda ),$$ (6)

where $P_{o}$ is the initial pressure distribution, Γ(r) is the Grüneisen parameter, H(r,λ) is the absorbed optical energy distribution. The absorbed optical energy inside the tissue medium can be expressed as^7^

$$H(r,\lambda )=\mu_{a}(r,\lambda )\phi (r,\lambda ),$$ (7)

where $\mu_{a}(r,\lambda )$ is the optical absorption coefficient of tissue medium, and ϕ(r,λ) is the optical fluence distribution in the tissue medium. The recorded pressure signal during the forward problem is then backpropagated in the time-reversed order with the Dirichlet boundary condition while performing time reversal (TR) reconstruction.^64^ Our main goal is to recover $\mu_{a}$ distribution more accurately from $P_{o}(r)$ using the DL fluence compensation method.

### 2.6 Deep Learning Formulation and DL Models

Optical fluence correction from the reconstructed OA images is a highly ill-posed problem, leading to inaccurate recovery of absorption coefficient due to the presence of noise, limited detector bandwidth, and unknown optical medium properties for fluence estimation. In TR reconstruction, the images are blurred and noisy due to the fluence corruption, discontinuous boundary condition, and electronic noise at the detector. The above problem could be tackled by using image postprocessing methods and thus improve the overall reconstructed image quality. The OA imaging formula can be rewritten as a DL inverse problem, which can be expressed as^66^

$$\overset{¯}{y}=Ax+\epsilon ,$$ (8)

where $\overset{¯}{y}\in R^{m}$ is measured data, $A\in R^{m\times m}$ operator indicating the fluence effect, $x\in R^{m}$ is optical absorption map, $\epsilon \in R^{m}$ is the noise. The fluence corrected image can be formulated as a supervised learning problem. In this context, the main aim is to find the nonlinear mapping function A:x↦y^ using paired training dataset $\left\{x^{\left(i\right)},y^{\left(i\right)}:i=1,2,3,\ldots ,n\right\}$. The fluence corrected images can be obtained by training a neural network to minimize the following loss function:^67^

minθ 1n∑i=1nL(y(i),y^(x(i),θ)), (9)

where n, θ, $y^{\left(i\right)}$, $x^{\left(i\right)}$, y^, indicate the number of training datasets, trainable parameters by the model, reference image, fluence corrupted image, and reconstructed image, respectively.

In our study, we have used different DL models, such as U network (U-Net), fully dense U network (FD U-Net), Y network (Y-Net), fully dense Y network (FD Y-Net), deep residual U network (deep ResUnet), and generative adversarial network (GAN). These networks follow different approaches to understand the feature vector with high-level embeddings inside the network architecture. In this study, we use end-to-end map training to remove the optical fluence effect from optoacoustic images and evaluated the performance of different types of DL models. The reason for choosing these models are outlined below:

U-Net: U-Net is designed to learn fluence effect over the entire field of view and correct for the same.

FD U-Net: In a dense block, earlier convolutional layers are connected to all subsequent layers via skip connection, which learns additional features from the input image. Since additional features are learnt via skip connection, it is excepted that FD-UNet would perform better fluence correction and provide images with improved quality.

Y-Net: Y-Net was designed to have two encoder networks, one of the encoder is used to extracts physical features from the raw sinogram data, whereas the second encoder network is used to extract texture features from image data. Since Y-Net architecture is being trained with both sinogram and image domain data, it is expected to perform accurate fluence correction than U-Net architecture.

FD Y-Net: A dense block is added upon the Y-Net model similar to FD-UNet, which will improve the semantics learnt for fluence correction. Note that the parameters are much larger than the previously explained networks.

Deep Res U-Net: Here, the skip connection between a residual unit and between low and high levels of the network will facilitate information propagation without degradation. Therefore, it reduces the number of training parameters (required in scenarios of limited memory), thereby reducing the training time and also improving the semantic learning.

GAN: All the above networks do not have adversarial training, hence a GAN based architecture was used to perform adversarial learning in the context of fluence correction. Since a generator and discriminator competition exists, this network has better learning capacity in terms of fluence correction in optoacoustic tomography. Specific architecture details used for U-Net, FD U-Net, Y-Net, FD Y-Net, Deep Res U-Net, and GAN are given in the Supplementary Material along with the architecture figures. The developed codes have been given as open-source for enthusiastic users in https://github.com/arumugarajm/Optical-Fluence-Removal-from-PAT-image.

### 2.7 Evaluation Metrics

To evaluate the model performance, all the model results are compared with the most common metrics such as peak signal-to-noise ratio (PSNR), structural similarity index measure (SSIM), and contrast to noise ratio (CNR).

#### 2.7.1 Peak signal-to-noise ratio (PSNR)

PSNR^68^ is used for measuring the quality of reconstructed image in dB based on mean squared error between the reconstructed image (μ^aij) and the reference image $\left(\mu_{\mathrm{aij}}\right)$^68^

$$\mathrm{PSNR}=20\;\log_{10}\;\frac{{\mathrm{MAX}}_{I}}{\sqrt{\mathrm{MSE}}},$$ (10)

where

MSE=1n2∑i=1n∑j=1n(μaij−μ^aij)2, (11)

where ${\mathrm{MAX}}_{I}$ indicates the maximum possible intensity value in the reconstructed image. The higher the PSNR, the better the reconstructed image quality.

#### 2.7.2 Structural similarity index measure (SSIM)

SSIM^69^ is used to compute the similarity between the output and the ground truth images. This value ranges between −1 and 1, a higher value indicates a better quality for output image and it can be mathematically expressed as^69^

SSIM(μa,μ^a)=(2μμaμμ^a+C1)+(2σμaμ^a+C2)(μμa2+μμ^a2+C1)(σμa2+σμ^a2+C2), (12)

where $\mu_{\mu_{a}}$ and $\mu_{{\hat{\mu }}_{a}}$ are the average of ground truth and the predicted images, respectively, $\sigma_{\mu_{a}}$ and $\sigma_{{\hat{\mu }}_{a}}$ indicate the variance of the ground truth and the predicted images, respectively, $\sigma_{\mu_{a}{\hat{\mu }}_{a}}$ indicates the covariance between the ground truth and predicted image, $C_{1}={\left(k_{1}L\right)}^{2}$ and $C_{2}={\left(k_{2}L\right)}^{2}$, where L is the dynamic range of the pixel values, $k_{1}=0.01$ and $k_{2}=0.03$.

#### 2.7.3 Generalized contrast to noise ratio (GCNR)

The gCNR^70^ is used to discriminate the target region from the background. Specifically, gCNR was shown to be a better metric compared with CNR in the context ultrasonography and OAI.^70^ The gCNR can be mathematically expressed as

gCNR=1−OVL. (13)

Here, overlapping area (OVL) is given as

$$\int \min\{p_{i}(x),p_{o}(x)\}dx,$$ (14)

where $p_{i}(x)$, $p_{o}(x)$ are the probability density function of the signal inside the target area, and outside the target area, respectively. N bins with centers at $\left\{x_{0},x_{1},\ldots ,x_{N-1}\right\}$ were used to obtain an equivalent histogram-based equation for the gCNR.^71^^,^^72^ For the target and background ROIs, the corresponding histograms, $h_{i}$ and $h_{o}$, were generated. Equation (13) was then discretized to produce the following equation:^71^^,^^72^

$$g\mathrm{CNR}=1-\overset{N-1}{\underset{k=0}{\sum }}\;\min\{h_{i}(x_{k}),h_{o}(x_{k})\},$$ (15)

where k is the index of the bin. Equation (15) was used to estimate the gCNR values (the foreground and background regions used are indicated in the corresponding figures).

#### 2.7.4 Contrast to noise ratio (CNR)

To evaluate the reconstruction performance of the different DL models, the contrast-to-noise ratio^70^ was used as a figure of merit, which can be expressed as

$$\mathrm{CNR}=\frac{\left|\mu_{\mathrm{roi}}-\mu_{\text{back}}\right|}{\sqrt{\sigma_{\mathrm{roi}}^{2}+\sigma_{\text{back}}^{2}}},$$ (16)

where μ and σ represent mean and variance of the region of interest and chosen background in the reconstructed image. Better image quality means a higher CNR value.

## 3 Deep Learning Implementation Details

The data-driven approach generally needs a large number of datasets for training the model and learning the features to generate relevant output from unseen data. Due to clinical constraints, we were unable to obtain large clinical datasets to train the DL models. Therefore, the DL models were trained with simulated dataset and we tested the trained model with both *in-vivo* and *in-silico* datasets. Our aim was to check if the DL models are capable of correcting for fluence effects, hence different DL architectures were trained with fluence corrupted images as input and ground truth as the reference output. Further, the performance of these different DL models was compared using the standard figure of metrics.

### 3.1 2D and 3D Data Generation for Training and Testing

Fundus datasets from different online repositories were accumulated such as: (1) 1000 fundus images with 39 categories from Kaggle^73^ and (2) 3200 images from retinal fundus multi-disease image dataset (RFMID).^74^ From the above-mentioned images, a few images did not support the vasculature extraction process (explained in Sec. 2.2), so we consider only those images supporting the vasculature extraction process. Totally, 2323 images were processed and further data augmentation was performed (one flipping, and two rotation operations were performed) to increase the total number of training data. After the data augmentation operation 9292 images were generated, 80% of images were used for training and the remaining images were used for validating (10%) and testing (10%) the models. For the case of *in-vivo* dataset: 60 images were used for testing (the model trained with vasculature dataset was used for testing *in-vivo* dataset).

3D numerical breast phantom datasets were collected from different online open-access links such as (1) optical and acoustic breast phantom database^75^ and (2) 3D acoustic numerical breast phantoms.^76^ Totally, we obtained seven 3D phantoms, out of seven, five 3D phantoms were used for training (having 12,288 slices, which includes data augmentation), one 3D phantom was used for validation, and one 3D phantom was used for testing. All the datasets were normalized between 0 and 1 before training and denormalized the data after training to the recover original $\mu_{a}$ distribution.

### 3.2 Deep Learning Implementation Details

All the DL models were implemented, trained, and tested on Python 3.7.9, TensorFlow 2.4.1. These models were run on Ubuntu 20.04 LTS system (Intel i9-9900K@3.60 GHz, 64 GB RAM, GeForce RTX 2070 SUPER). To train the DL model, we have used a normalized root mean square error (NRMSE) as the loss function; mathematically it can be expressed as

NRMSE(y,y^)=1n∑i=1n(yi−y^i)21n∑i=1n(yi)2, (17)

where $y_{i}$ and ${\hat{y}}_{i}$ represent reference image, reconstructed image in the DL model, respectively. The numerator term is used to measure the pixel-wise differences between the reference and reconstructed image, this pixel-wise difference is averaged over the entire image; square root is taken of the resultant to obtain a Euclidean distance measure. The Euclidean distance is then normalized by dividing it with respect to the L2-norm of the reference image. Note that the Euclidean distance will be small if the reconstructed images are very close to the reference images. Further, if the reconstructed and reference images differ substantially the Euclidean distance will be large. Normalizing will facilitate the comparison between datasets or models at different scales. We trained the models for 150 epochs, Adam optimizer was used with a learning rate of $1\times e^{-4}$ with a batch size of 6 images. We used early stopping with patience as 12, which stops the training process when there is no further reduction in the loss function.

## 4 Results

**Fig. 3:**
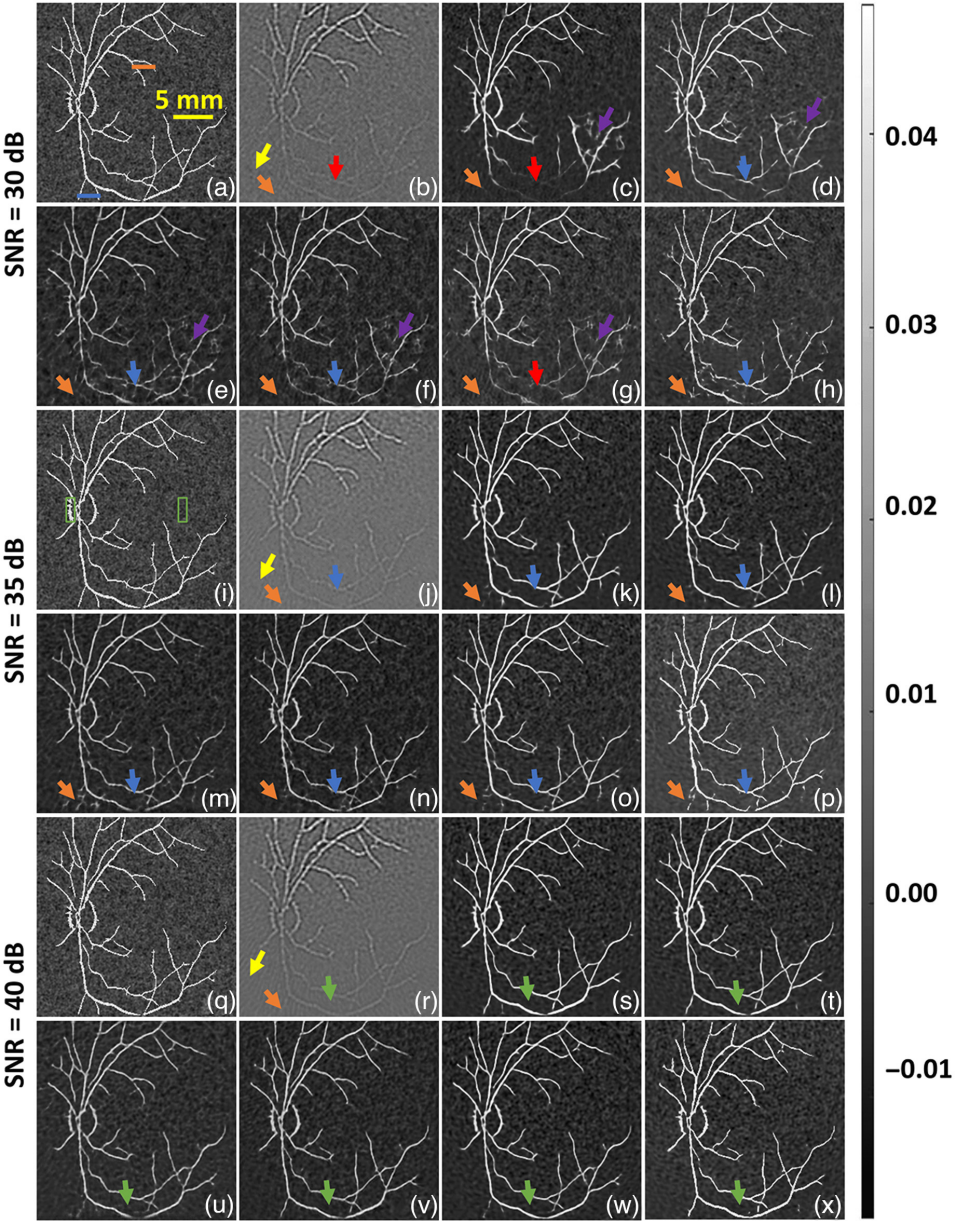
Qualitative comparison of fluence correction using digital vasculature phantom with different DL models. (a), (i), (q) Reference image. (b), (j), (r) TR reconstruction OA image. (c), (k), (s) U-Net result. (d), (l), (t) FD U-Net result. (e), (m), (u) Y-Net result. (f), (n), (v) FD Y-Net result. (g), (o), (w) Deep ResUnet result. (h), (p), (x) GAN result. Panels (a)–(h) correspond to ${\mathrm{SNR}}_{d}=30\;\mathrm{dB}$, (i)–(p) correspond to ${\mathrm{SNR}}_{d}=35\;\mathrm{dB}$, and (q)–(x) correspond to ${\mathrm{SNR}}_{d}=40\;\mathrm{dB}$. Different colored arrows are used to indicate the fluence effect and the improvement obtained using DL methods. The red arrow indicates deep vasculature, which was not visible due to the fluence effect; the yellow arrow indicates artifacts in the image; the orange arrow shows the inability to recover the deep vasculature; the purple arrows indicate undesirable reconstruction; the blue arrow shows slight improvement in the deep vasculature region; and the green arrow shows good improvement in the deep vasculature. The orange and blue lines in (a) were used to draw line plots at shallow and deeper region respectively. The green boxes shown in (i) were used to indicate foreground and background regions to calculate gCNR.

Initially, we evaluated all the DL models with a 2D BV phantom with nonhomogeneous background, these numerical experiments were considered at a wavelength of 700 nm has different data noise levels, i.e., ${\mathrm{SNR}}_{d}=30$, 35, and 40 dB SNR. Figure 3(a) shows the reference ground truth image and Fig. 3(b) shows the TR reconstructed image with data noise level of 30 dB, the yellow arrow in Fig. 3(b) indicates the reconstruction artifacts, the red arrow highlights the misrepresentation of deep vasculature, and the orange arrow indicates the effect of fluence on small vasculature at deep tissue region. In all DL models, some undesirable vasculature (indicated by a purple arrow) appears on the reconstructed images. Figures 3(c)–3(h) show the reconstruction results corresponding to U-Net, FD U-Net, Y-Net, FD Y-Net, Deep ResUnet, and GAN, respectively. Figures 3(c)–3(h) illustrate that all the DL models have the ability to recover the shape of the vasculature in the deeper region, but the recovered vasculature seems to have discontinuity [indicated by red arrows in Figs. 3(c)–3(g)]. This discontinuity seems to be much less using the GAN model as seen in Fig. 3(h) with 30 dB noise. Further, as the data noise is reduced, i.e., ${\mathrm{SNR}}_{d}=35\;\mathrm{dB}$, we observe that the discontinuity in the deep vasculature pattern reduces as indicated by blue arrows in Figs. 3(k)–3(p). As the data noise is reduced, the fluence correction using DL models seems to become more accurate as indicated by blue arrow (wherein the vasculature is becoming more continuous) and further the artifact seems to be eliminated by DL models. Figures 3(s)–3(x) indicate the fluence corrected images using different DL models with all ${\mathrm{SNR}}_{d}=40\;\mathrm{dB}$. As expected the fluence corrected images after DL model seems to more closely matching the ground truth with increased image quality. Note that for all these cases, training and testing were performed at 700 nm.

**Fig. 4:**
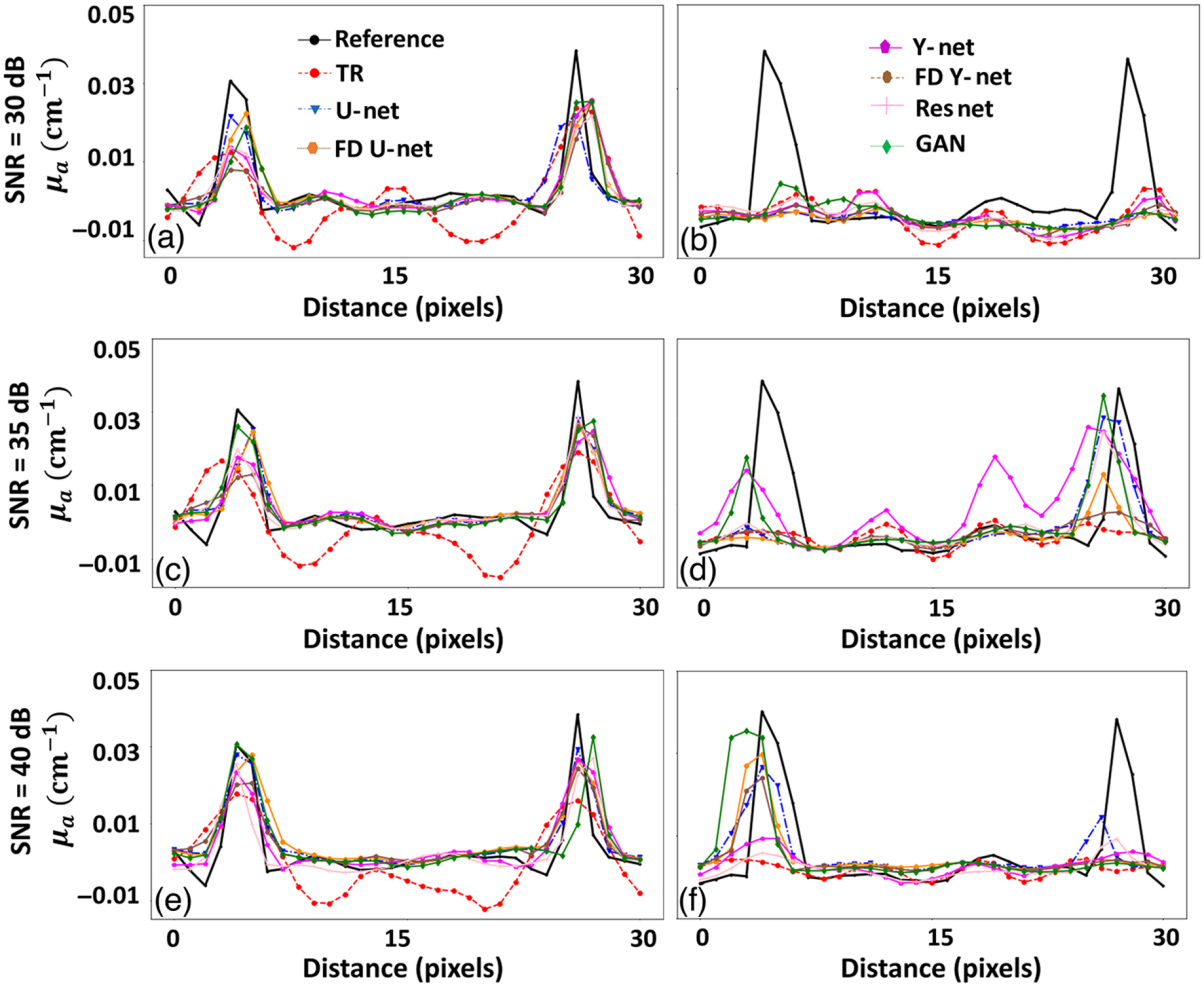
Line profiles along the orange and blue lines are shown in Fig. 3(a). (a) Line plot along the orange line with ${\mathrm{SNR}}_{d}=30\;\mathrm{dB}$, (b) line plot along the blue line with ${\mathrm{SNR}}_{d}=30\;\mathrm{dB}$, (c) line plot along the orange line with ${\mathrm{SNR}}_{d}=35\;\mathrm{dB}$, (d) line plot along the blue line with ${\mathrm{SNR}}_{d}=35\;\mathrm{dB}$, (e) line plot along the orange line with ${\mathrm{SNR}}_{d}=40\;\mathrm{dB}$, and (f) line plot along the blue line with ${\mathrm{SNR}}_{d}=40\;\mathrm{dB}$.

The line plot corresponding to all the results shown in Fig. 3 (close to the source and away from the laser source) is illustrated in Fig. 4. Line profiles clearly indicates that the efficacy of the DL models to perform fluence correction is higher in less noise cases, i.e., ${\mathrm{SNR}}_{d}=40\;\mathrm{dB}$ than the high noise case, i.e., ${\mathrm{SNR}}_{d}=30\;\mathrm{dB}$. The line plots at the superficial region show that the DL model improves the accuracy over the TR reconstruction, and in deeper region DL models like FD U-Net and GAN seems to outperform other DL models. Quantitative results corresponding to Figs. 3 and 4 are given in Table 1. In all noise levels, FD U-Net seems to provide the best SSIM value. Further, the DL models seem to improve over the traditional reconstruction scheme by >10% in terms of PSNR. In essence, it can be noted that DL models seem to perform accurate fluence correction when the data noise is lesser.

**Table 1:** Quantitative comparison for effect of noise on DL based fluence correction. The bold font in the table denotes the best PSNR and SSIM values.

Network	Set 1 (30 dB SNR)		Set 2 (35 dB SNR)		Set 3 (40 dB SNR)	
	PSNR	SSIM	PSNR	SSIM	PSNR	SSIM
Input/TR	40.340	0.838	40.654	0.854	40.702	0.857
U-Net	42.925	0.920	**44.629**	**0.951**	44.909	0.959
FD U-Net	**43.479**	**0.931**	44.567	**0.951**	**45.089**	0.959
Y-Net	43.118	0.923	44.358	0.948	45.055	**0.960**
FD Y-Net	43.187	0.922	44.257	0.943	44.835	0.944
Deep residual Net	43.278	0.925	44.556	0.949	**45.089**	0.959
GAN	43.335	0.928	43.886	0.947	44.330	0.954

**Fig. 5:**
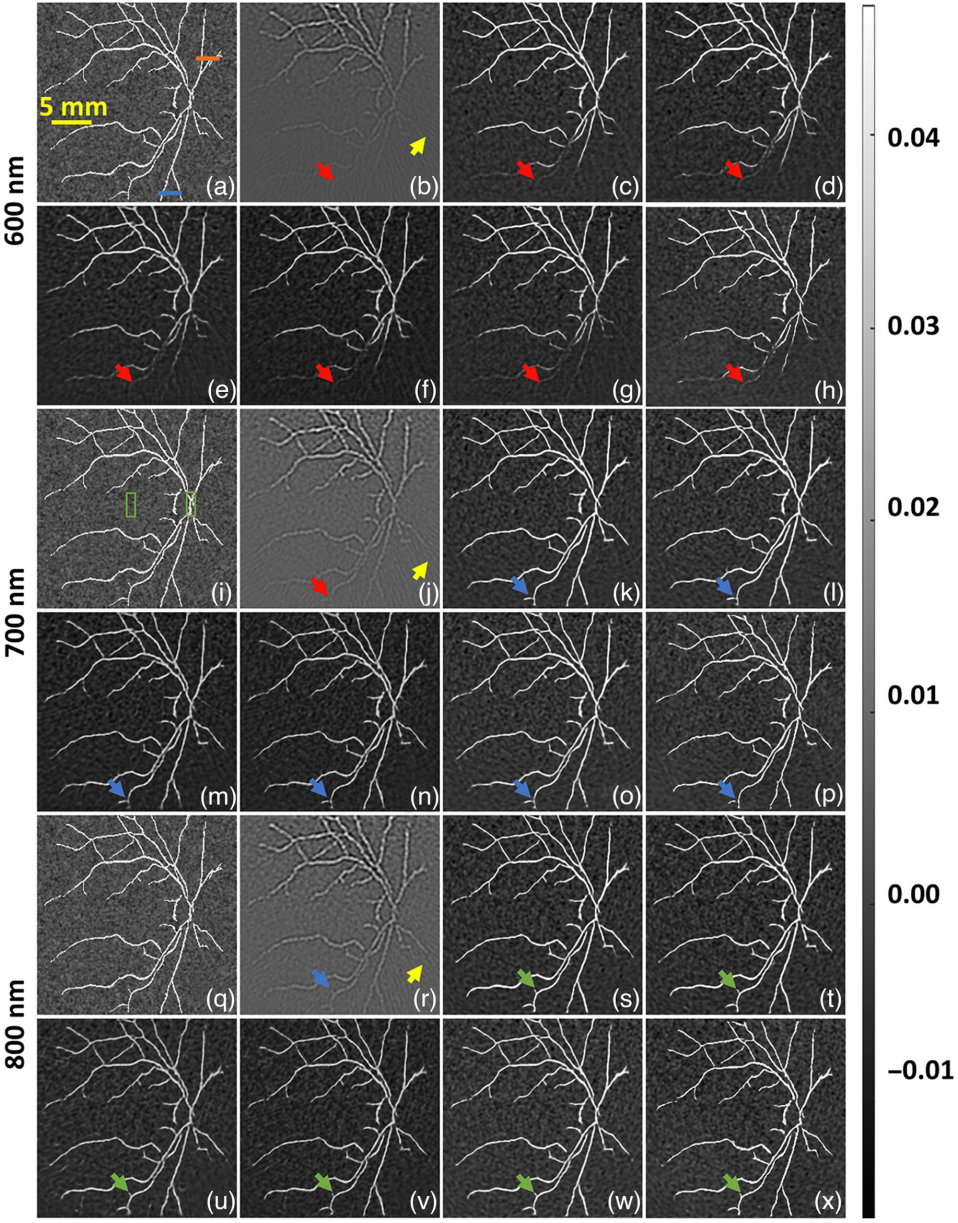
Qualitative comparison of fluence correction using digital vasculature phantom with different DL models. (a), (i), (q) Reference image. (b), (j), (r) TR reconstruction OA image. (c), (k), (s) U-net result. (d), (l), (t) FD U-Net result. (e), (m), (u) Y-Net result. (f), (n), (v) FD Y-Net result. (g), (o), (w) Deep ResUnet result. (h), (p), (x) GAN result. Panels (a)–(h) correspond to a wavelength of 600 nm, (i)–(p) correspond to a wavelength of 700 nm, and (q)–(x) correspond to a wavelength of 800 nm. Different colored arrows are used to indicate the fluence effect and its improvement in the recovered images using DL methods. The red arrows show deep vasculature, which was not visible due to the fluence effect; the yellow arrow indicates artifacts in the image; the blue arrow shows improvement in the deep vasculature region; and the green arrows are used to indicate improvements in the deep vasculature region. The orange and blue lines in (a) were used to draw line plots at shallow and deeper region respectively. The green boxes shown in (i) were used to indicate foreground and background regions to calculate gCNR.

It is well known that the depth-dependent fluence effect is heavily influenced by wavelength. Hence, we have analyzed the effect of wavelength on the reconstructed images and fluence correction approach using DL models. We have considered three different wavelengths (600, 700, and 800 nm) and checked the performance of the fluence correction using DL models as shown in Fig. 5. Herein, the first two rows represent the 600-nm case, the next two rows indicate the results using 700 nm and the last two rows correspond to 800-nm case. In all the cases the DL model was trained with the images generated at a wavelength of 700 nm. When we used a lower wavelength, i.e., 600 nm compared to the training set, it can be clearly seen that the deep vasculature structure is more distorted compared to 700 nm [compare Fig. 5(b) with Fig. 5(j)]. This occurs because of higher optical absorption and scattering effect at a lower wavelength (600 nm) compared to a higher wavelength (700 nm).^15^ We wanted to see if the trained DL model at a particular wavelength for fluence correction could be adapted to other wavelength regimes.

Figures 5(c)–5(h) indicate the recovery of tissue vasculature at 600 nm, the DL models were able to perform fluence correction. However vasculature present at larger depths was not recoverable due to very limited signal in these regions as indicated by the red arrow in Figs. 5(b)–5(h). Along the same lines, when fluence correction was performed using DL models at 700 and 800 nm, the fluence correction seems to be very accurate as indicated by blue arrows in Figs. 5(k)–5(p) and green arrows in Figs. 5(s)–5(x). Overall, it can be concluded that DL models could be used for fluence correction, wherein the DL model is trained at one of the wavelengths and testing is performed at another wavelength. However, as was seen the performance at 600 nm (lower wavelength) was suboptimal due to limited signal reaching the deeper region. In this study we observed that increasing the wavelength in NIR-I window increased the light penetration, resulting in higher contrast in the image and better fluence correction using a DL model.

**Fig. 6:**
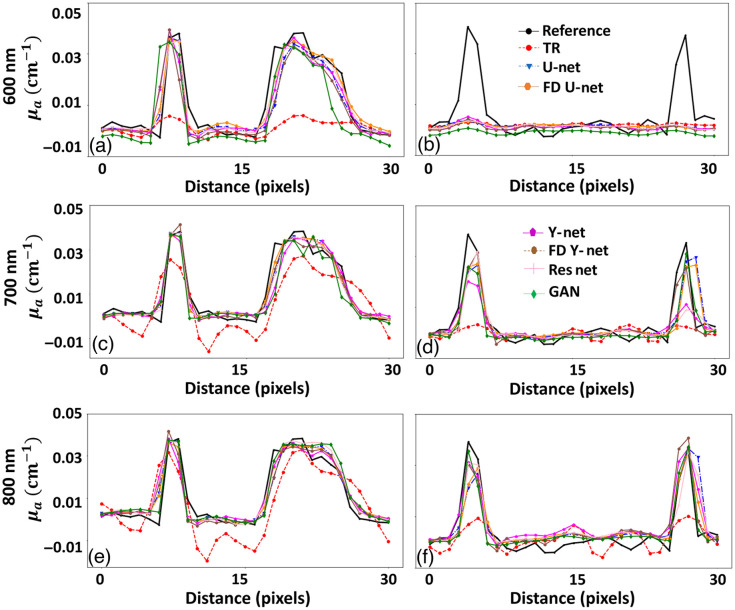
Line profiles along the orange and blue lines indicated in Fig. 5(a). (a) Line plot along the orange line with λ=600  nm, (b) line plot along the blue line with λ=600  nm, (c) line plot along the orange line with λ=700  nm, (d) line plot along the blue line with λ=700  nm, (e) line plot along the orange line with λ=800  nm, and (f) line plot along the blue line with λ=800  nm.

To check the performance of DL-based fluence compensation and accurate recovery of the $\mu_{a}$ distribution at different wavelengths, we plotted the two line profiles as shown in Fig. 6 along the orange and blue lines indicated in Fig. 5. The first, second, and third rows in Fig. 6 correspond to the 600, 700, and 800 nm cases, respectively. Figures 6(a), 6(c), and 6(e) indicate that fluence effects could be corrected using the different DL models very accurately, with the accuracy being higher for 700 and 800 nm cases as opposed to 600 nm case. Figures 6(d) and 6(f) indicate that accurate recovery of deeper vasculature is possible in both 700 and 800 nm cases with FD Y-Net and FD U-Net performing the best. However as expected, Fig. 6(b) indicates that the fluence correction for 600 nm case is suboptimal, and the different DL models fail to improve upon the reconstructed image quality. This is primarily due to lesser signal strength in the reconstructed TR images. In essence, we can conclude that the fluence correction accuracy across all the wavelengths for superficial and intermediate depths is higher compared to the deeper vasculature region (owing to signal strength at that particular wavelength). Quantitative comparisons using PSNR, and SSIM are given in Table 2, as can be seen, the FD U-Net outperforms other DL models in terms of fluence correction.

**Table 2:** Quantitative comparison of the effect of wavelength on DL based fluence correction. The bold font in the table denotes the best PSNR and SSIM values.

Network	Set 4 (600 nm)		Set 5 (700 nm)		Set 6 (800 nm)	
	PSNR	SSIM	PSNR	SSIM	PSNR	SSIM
Input/TR	39.969	0.832	40.830	0.860	37.913	0.802
U-Net	42.014	0.888	45.610	0.964	45.708	0.967
FD U-Net	42.081	0.878	**45.731**	**0.965**	**45.834**	**0.968**
Y-Net	42.255	0.889	44.725	0.958	45.575	0.966
FD Y-Net	41.976	0.867	45.076	0.960	45.459	0.965
Deep residual Net	41.976	0.863	45.143	0.960	45.399	0.965
GAN	**42.916**	**0.921**	44.756	0.958	45.685	0.963

A quantitative comparison of vascular detectability for different noise and wavelength variation using gCNR metric is given in Table 3. gCNR indicates that FD U-Net outperforms most of the other networks for fluence compensation. Further the recovered contrast of deep vasculature structure seems to be much higher compared to TR reconstruction.

**Table 3:** Quantitative comparison of vascular detectability for different noise and wavelength values. The bold font in the table denotes the best gCNR values for different sets.

Network	Set 1 (30 dB SNR)	Set 2 (35 dB SNR)	Set 3 (40 dB SNR)	Set 4 (600 nm)	Set 5 (700 nm)	Set 6 (800 nm)
	gCNR	gCNR	gCNR	gCNR	gCNR	gCNR
Input	0.596	0.654	0.735	0.511	0.606	0.703
U-Net	0.829	0.850	0.872	0.783	0.838	0.860
FD U-Net	**0.845**	**0.857**	**0.881**	0.798	**0.856**	**0.871**
Y-Net	0.832	0.849	0.880	0.798	0.847	0.864
FD Y-Net	0.835	0.855	0.877	0.770	0.847	0.861
Deep residual Net	0.823	0.847	0.861	0.750	0.839	0.857
GAN	0.841	0.843	0.854	**0.810**	0.845	0.853

OAI has shown great promise in breast imaging,^77^ hence we have performed a volumetric 3D fluence correction using numerical breast phantom. Due to the limited number of available 3D datasets, we trained and tested all the DL models with 2D slices (X–Y slices) and then the tested 2D slices were stacked together to form a 3D volume. We have reported the results using the different DL models in both 3D and different 2D projection views (X–Y, Y–Z, X–Z). Note that the fluence effect, i.e., the light propagation model, was run in a fully 3D setting.

**Fig. 7:**
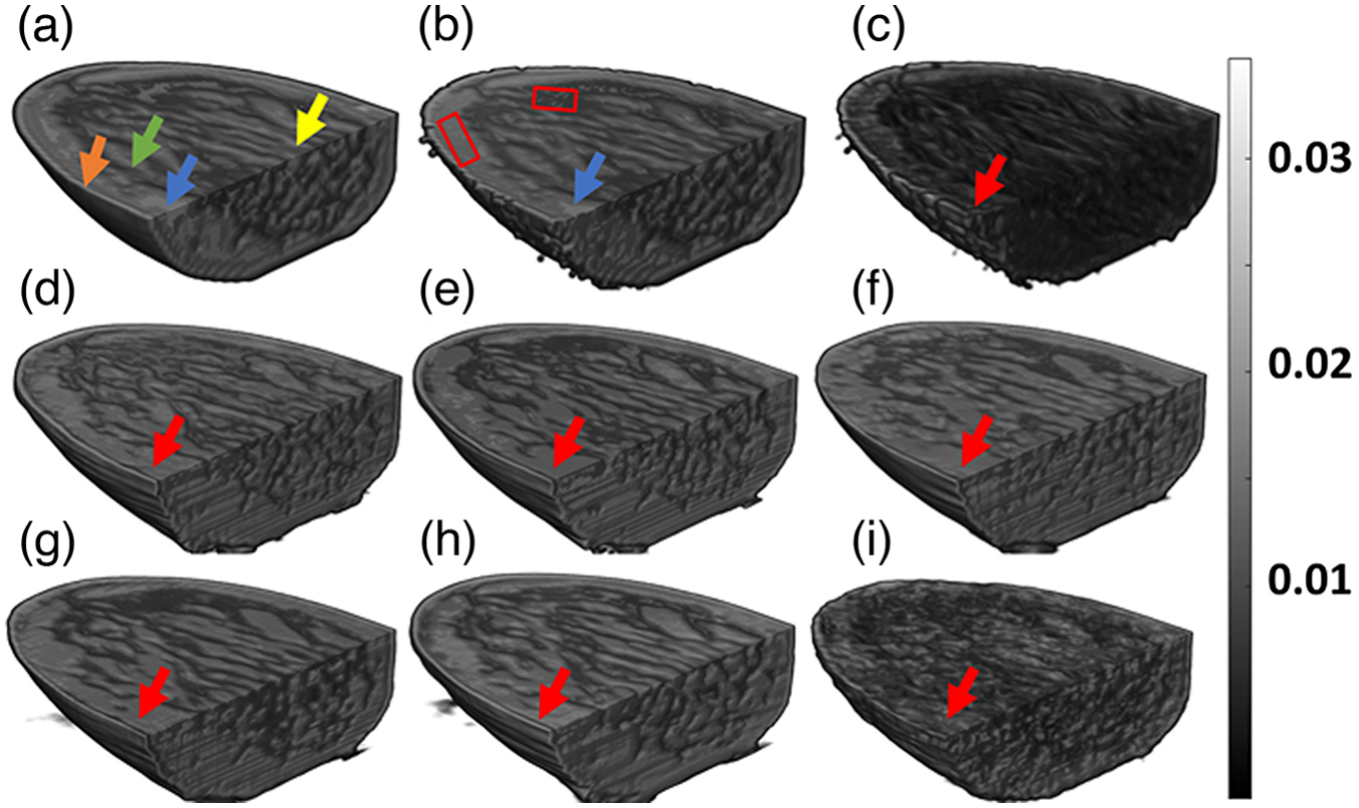
Cross-sectional slice view of 3D numerical breast phantom data. (a) Ground truth, (b) fluence affected volume, (c) TR, (d) U-Net-based fluence correction, (e) FD U-Net-based fluence correction, (f) Y-Net-based fluence correction, (g) FD Y-Net-based fluence correction, (h) Res Net-based fluence correction, and (i) GAN-based fluence correction. Yellow, blue, green and orange arrows represent fat, vessle, fibroglandular, and skin, respectively. The red arrows indicate the fluence effect in the blood vessel. The red boxes are used to represent the fluence effect in the breast tissue.

For better visualization of 3D fluence correction results, we have sliced the 3D volume data, and as shown in Fig. 7. Figure 7(a) is the ground truth volume and the arrows with different color indicate different tissue types that are typically present in the breast. Figure 7(b) shows the fluence affected 3D volume, the vessel close to the boundary is clearly visible as indicated by the blue arrow, however, deeper tissue regions have lost contrast as shown by rectangular boxes in Fig. 7(b). Figure 7(c) indicates the TR reconstructed volume, wherein the different tissue types are not reconstructed accurately except for the skin lining. Figures 7(d)–7(i) indicate the 3D fluence corrected volumes obtained using the different DL models. All the different tissue regions were recovered accurately using DL models, specifically the regions indicated by rectangular boxes in Fig. 7(b). As shown by red arrows, the DL models were unable to recover the vessel, because the X−Y slice had smaller vessels, which are difficult to be captured by the DL models.

**Fig. 8:**
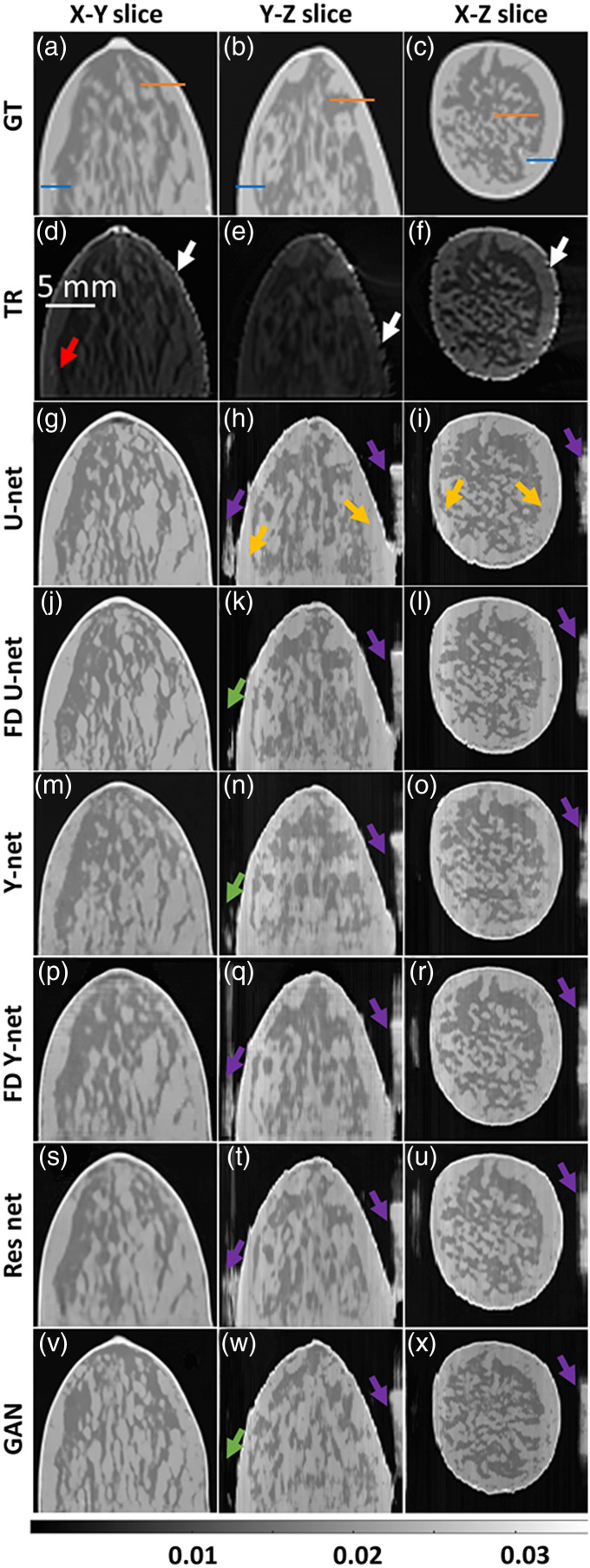
Visual comparison along different 2D planes using the various DL-based fluence correction models. The models were trained and tested with 750-nm wavelength and the data was having 40-dB SNR. Different colored arrows used to indicate the fluence effect and the improvements using DL methods. The red arrow is used to indicate the fluence effect, the white arrow shows destruction of boundary, the purple arrow represents undesirable structures in the image, the gold arrow shows line artifacts, and the green arrow indicates improvement in the reconstruction quality. Solid orange and blue line lines were used to draw line plots at different locations shown in (a)–(c).

The visual comparison of fluence correction for numerical breast phantom with different slices is shown in Fig. 8. Here, columns indicate different planes, and rows represent the ground truth, the TR, and the different DL-based fluence correction methods. From Fig. 8(d), the TR reconstructed images are unable to recover the vessels as indicated by red arrow due to fluence effect. The destruction of shape (as indicated by white arrow in the second row) was recovered by all the DL models. Note that the DL-based fluence correction improved the image quality drastically, however, the networks were unable to recover the small vasculature. From Figs. 8(g)–8(x), it can be noticed that X–Y slices were accurately corrected as opposed to Y–Z or X–Z slices, which might be due to training the models using X–Y slices. Few undesirable artifacts seem to arise in the Y–Z and X–Z slices as indicated by purple and gold arrows. Note that the severity of these artifacts is lesser while using FD U-Net, Y-Net, and GAN networks compared with other networks, which are indicated by green arrows. Figure 8 clearly illustrates that FD U-Net is the best model for fluence correction. In addition, the quantitative evaluation of breast numerical phantom is shown in Table 4. The quantitative comparison was performed using the whole 3D volume and different planar views. A 5 to 6 dB improvement was observed in terms of PSNR values and about 9% improvement was noticed in SSIM values for volumetric comparison. Along the X–Y, Y–Z, and X–Z planes, the improvements in PSNR value are ∼8 to 10 dB, 4.4 to 4.6 dB, 5.5 to 6 dB, respectively. Similarly, the SSIM values were found to increase by 17.6% to 18%, 9% to 12.2%, 11% to 12.9% along X–Y, Y–Z, and X–Z planes, respectively. Line plots along each of planes are given in the Supplementary Material. Overall, it was observed that DL models have great potential in correcting for fluence in 3D settings.

**Table 4:** Quantitative comparison of 3D volumetric data of different 2D planar slices. The numeric values are represented as average ± standard deviation. The bold font in the table denotes the best PSNR and SSIM values.

Network	X–Y slices		Y–Z slices		X–Z slices	
	PSNR	SSIM	PSNR	SSIM	PSNR	SSIM
Input/TR	39.064 ± 0.066	0.799 ± 0.005	39.603 ± 0.091	0.813 ± 0.005	39.445 ± 0.415	0.812 ± 0.015
U-Net	49.475 ± 0.254	**0.980 ± 0.002**	43.854 ± 0.555	0.922 ± 0.008	45.465 ± 0.345	0.941± 0.002
FD U-Net	**49.770 ± 00258**	**0.980 ± 0.002**	44.280 ± 0.262	**0.935 ± 0.002**	45.495 ± 0.073	**0.947 ± 0.001**
Res Net	49.099 ± 0.491	0.975 ± 0.001	43.532 ± 0.476	0.913 ± 0.007	45.358 ± 0.096	0.934 ± 0.001
Y-Net	48.777 ± 0.621	0.978 ± 0.002	44.022 ± 0.142	0.926 ± 0.002	45.044 ± 0.140	0.943 ± 0.002
FD Y-Net	48.599 ± 0.697	0.977 ± 0.001	43.271 ± 0.290	0.903 ± 0.007	44.952 ± 0.387	0.929 ± 0.006
GAN	49.006 ± 0.323	0.976 ± 0.002	**44.768 ± 0.290**	0.933 ± 0.003	**45.712 ± 0.154**	**0.947 ± 0.003**

**Fig. 9:**
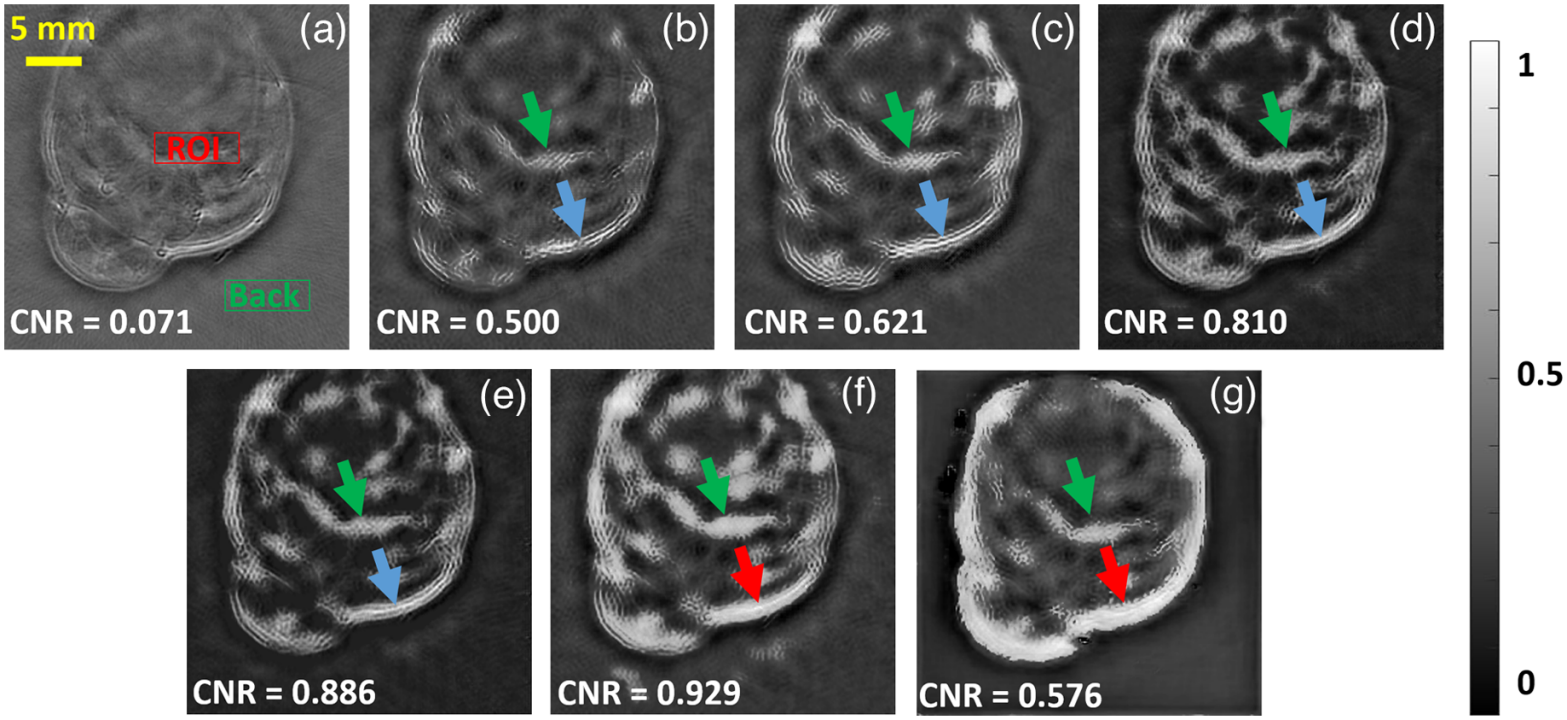
Testing result for *in-vivo* mice dataset. (a) Conventional reconstruction image, (b) U-Net based fluence correction, (c) FD U-Net based fluence correction, (d) Y-Net based fluence correction, (e) FD Y-Net based fluence correction, (f) deep ResUnet-based fluence correction, and (g) GAN-based fluence correction. The intensity value is normalized between 0 and 1. Different colored arrows used to indicate the fluence effect and its improvement using DL methods. The green arrows show the contrast variations obtained using different methods, the red arrow indicates destruction of boundaries, the blue arrows are used to indicate improvements in the reconstruction.

To evaluate the feasibility of the DL models for fluence correction, we tested all the DL models with MSOT scanned mice datasets (*in-vivo*) and the results are shown in Fig. 9, the data acquisition is explained elsewhere in Ref. 4. Procedures involving animal experiments were approved by the Animal Care and Handling Office of Helmholtz Zentrum München and the Government of Upper Bavaria. For the *in-vivo* dataset, domain translation was used, wherein the DL models were trained using vasculature datasets and testing was performed using *in-vivo* dataset. Similar to *in-silico* data, the DL models were able to perform fluence correction operation on the reconstructed images with *in-vivo* mice data, thereby it can improve the contrast of the image. The blue arrows in the fluence corrected images indicate that the vasculature is recovered properly with *in-vivo* data, however, the Deep ResUnet and GAN have destroyed this vasculature, which is indicated by the red arrow. The center region of the mice is seen to have higher contrast with the DL-based fluence correction methods compared to the backprojection reconstruction, the same is highlighted by green arrows. Note that the CNR value is more, using the Deep ResUnet; however, visually, the vasculature seems to be destroyed and resolution has reduced. FD Y-Net is found to generate the best result, visually, and to have higher CNR as well. The *in-vivo* results suggest that DL models can perform accurate fluence correction using end-to-end learning thereby improving the image quality.

**Fig. 10:**
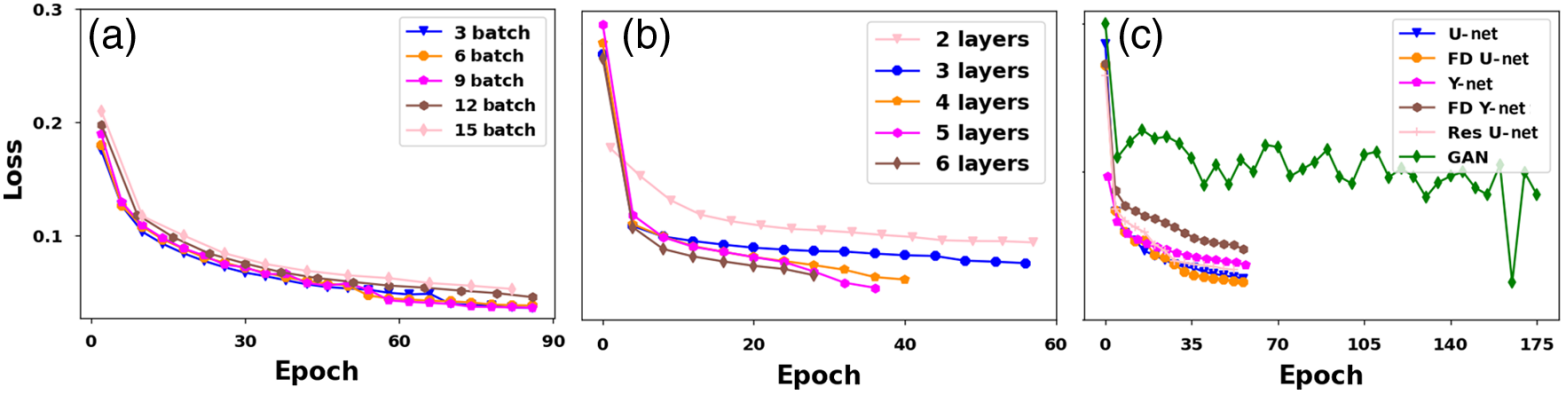
Ablation studies on DL-based fluence correction; (a) represents the effect of batch size in DL models, (b) represents the effect of depth in DL models, and (c) represents the loss curve for the different DL models.

The different parameters of the DL models like the number of layers/depth, batch size, and convergence analysis were studied in detail. Initially, we varied the batch size during training and as reported in Fig. 10(a). Figure 10(a) shows that the batch size indeed has an effect on the training loss, larger batch size increases the memory requirement and the computational cost too. On the other hand, lower batch size quickly updates the algorithm and generalizes the model very well. In all the considered network, we have chosen the batch size to be six to enable effective computation and memory allocation. The number of layers in DL models has a tradeoff between the computational cost and accurate fluence correction, as shown in Fig. 10(b). The number of trainable parameters (training time) is directly proportional to the number of layers. An accurate model would need more trainable parameters to result in good training. Hence we varied the number of layers in the DL network and found that having four layers is sufficient to enable accurate fluence correction (increasing layers further had marginal improvement in the loss). Therefore, all the DL models were trained with four layers only.

We also tested the convergence of the different models considered in this work, and the same is shown in Fig. 10(c). Figure 10(c) indicates that all the DL models are converging after certain epochs, and we concluded that about 60 epochs are sufficient for all the models except GAN. A GAN model generates plausible output after understanding the distribution of input samples, hence there is a lot of fluctuation while plotting the generator loss function and GAN required a lot of epochs to converge. We found 165 epoch had very less loss and hence 165 epoch was used for testing the GAN model. The FD U-Net was found to have lower cost function values. Hence as expected, FD U-Net was found to generate the best result for both 2D and 3D models in terms of PSNR and SSIM values among all the considered DL models. The number of trainable and nontrainable parameters along with training/testing time is shown in Table 5.

**Table 5:** Number of network training parameters, training and testing time for the different DL models for 3D breast phantom data.

Network	Trainable parameters	Nontrainable parameters	Training time (h:min)	Testing time (s)
U-Net	77,75,681	6,912	05:15	13.48
FD U-Net	3,00,65,601	30,464	07:22	26.08
Y-Net	94,07,275	7,552	09:10	19.50
FD Y-Net	3,64,40,107	37,760	11:52	41.94
Deep Res-Net	47,15,441	7,296	04:01	17.49
GAN	5,65,63,009	13,69,84,129	26:13	24.25

## 5 Discussion

The fluence correction method was tested in the context of 2D and 3D, wherein illumination geometry was varied; line illumination was achieved by having multiple point sources in the case of 2D imaging, whereas the point sources were placed on irregular boundary for the 3D imaging scenario. The model was trained with images reconstructed at 700 nm and 40 dB noise; this trained model was tested with the image obtained at 600 and 800 nm, having 30 and 35 dB data noise levels. Specifically for the 2D case, we can see Figs. 3 and 5 highlight the deep vasculature, which was not visible due to the fluence effect (indicated by red arrows). Further DL results were found to report superior image quality measures in terms of PSNR and SSIM. The 3D imaging result is shown in Fig. 8, the TR reconstruction results have been heavily influence by the fluence effect and are not close to ground truth (indicated by red arrow). On the other hand, the DL model compensates for the fluence effect and deep tissue can be clearly identified (as indicated by green arrows). Further, the fluence compensated results are closer to the ground truth compared with the TR reconstructed images indicating that DL indeed holds promise for fluence compensation. The 2D (Figs. 3 and 5; Tables 1 and 2), 3D (Fig. 8 and Table 3), and mice data (Fig. 9) results clearly indicate the DL models have great ability to perform fluence correction/improvement (indicated by green arrow), and among the different models being considered FD U-Net seems to perform well. Note that all the DL models were trained with optimal parameters (depth, batch size, and epochs) to enable a fair comparison. As opposed to earlier work wherein optical properties are assumed to correct for fluence,^16^^,^^20^^,^^23^^–^^28^ in this work the DL model automatically learns the effect of fluence and corrects for the same. This eliminates the bias of using optical properties from literature. Earlier works have used machine-learning models for fluence compensation, however, this was restricted to 2D imaging geometry. Our work has used DL models to perform fluence compensation in 3D imaging geometries. Further, the DL models eliminate the need for running computationally complex forward model based on DE or Monte Carlo. The DL-based fluence correction is about 17 times faster compared to the running forward model in NIRFAST.

One of the major problems with DL-based methods lies in their inability to generalize unseen data. Herein, we have trained the model using *in-silico* data and tested with *in-vivo* data. We found that such translation leads to a reduction in accuracy (black spots in the *in-vivo* results). Further, when testing the *in-vivo* data with DL models trained with breast phantom data, we observed that the *in-vivo* fluence correction was not realistic in nature. Therefore, the future work will focus on developing neural networks that can learn directly from *in-vivo* data using a model-based approach in an iterative fashion. Also, the DL models seem to be scalable and work at different noise levels and different wavelengths. Note that this work performed fluence correction with two different illumination geometry, i.e., a line illumination for 2D vascular network and along the entire breast (along a circle) for 3D breast data. For both these geometries, the DL-based fluence correction seems to work accurately.

The future work will focus on evaluating the performance of the developed DL models to perform accurate spectral unmixing. Further for the 3D case, the DL models were performing fluence correction slice-by-slice due to unavailability of large 3D datasets. The future work will focus on developing fully 3D DL models, which are computationally efficient at the same time develop methodology for generating large 3D datasets for training the DL models. Further deploying these models with 3D experimental datasets will also be evaluated. The fluence correction heavily depends on the illumination geometry, number of illumination sources, and the field of view; the effect of these different parameters on fluence correction will also be taken up in the future. In this work, we focused on optical heterogeneity and did not consider acoustic heterogeneity since we specifically wanted to evaluate the performance of DL methods for fluence compensation (which contributes a lot for multispectral optoacoustic tomography). Acoustic heterogeneity does play an important role, and it is important to develop a generalized DL model that will compensate for all the errors, which will be taken up as future work.

## 6 Conclusion

Different data-driven models were evaluated to perform fluence compensation in quantitative OAI. As opposed to earlier works, we have evaluated models on a highly optically heterogeneous medium having realistic optical properties. Further, the fluence compensation was performed both in 2D and 3D settings. The results indicated that the different DL models were adaptable/robust to noise in the data. Further, the DL models were able to perform superior fluence compensation at large wavelengths (compared to the wavelength used for training the network) as opposed to shorter wavelengths. All the DL models were effective in compensating for the light fluence effect with FD U-Net showing the best performance. For the 3D volumes, an 10% to 15% improvement was observed with the PSNR and SSIM metric, and for *in-vivo* dataset, an improvement by a factor of 10 times was observed in terms of CNR compared to standard TR reconstruction. Finally, the DL-based fluence correction model was found to be about 17 times faster compared to solving DE-based fluence compensation.

## Code, Data, and Materials Availability

The data that support the findings of this study are available from the corresponding author upon reasonable request. The codes are posted at https://github.com/arumugarajm/Optical-Fluence-Removal-from-PAT-image.
