# Supplementary material for: Deep learning methods hold promise for light fluence compensation in three-dimensional optoacoustic imaging
Source: J Biomed Opt. 2022 Oct 8;27(10):106004. doi: 10.1117/1.JBO.27.10.106004 (PMC9547608; doi:10.1117/1.JBO.27.10.106004)
Supplement: Supplementary file 1 [file JBO_027_106004_SD001.pdf]

Supplementary for Deep learning methods holds promise for light fluence compensation in three-dimensional optoacoustic imaging

Arumugaraj M[a], Vipul Gujrati[b], Vasilis Ntziachristos[b], Jaya Prakash*[a]
[a]Department of Instrumentation and Applied Physics, Indian Institute of Science, Bengaluru - 560 012
[b]Institute of Biological and Medical Imaging, Building 56, Ingolstädter Landstr. 1, D-85764, Neuherberg, Germany

DL model architectrues and line plot for 3D output results:

1 Network Architectures

In the subsequent section, we have explained the implementation details of the network architectures. All the DL network architectures which were used for our study are given here, Figs. 1-7.

1.1 U-Net and FD U-Net architecture

The original U-Net architecture was modified and used for fluence correction. The standard U-Net has two paths involving down-sampling and up-sampling operations. The encoder path repeatedly uses two 3×3 convolution layer with zero padding . Each convolution layers incorporates a batch normalization (BN), and a rectified linear unit (ReLU) activation function. In the down-sampling path, the spatial dimension of the input image is reduced by using a 2×2 max pooling operation with a stride 2. The number of channels are doubled after the pooling operations in each down-sampling layer. The decoder path repeatedly uses 2×2 up-sampling layer (transpose convolution layer) to increase the spatial dimensions of the image followed by a two 3×3 convolution and half the number of channels after the convolution operation. At each step, features extracted from the contracting path by convolution operation are concatenated with expansive path before performing the convolution operation. In the last layer, a 1×1 convolutional layer is used to reduce the final layer to a single channel and produce the predicted image. The used architecture is shown in Fig. 1.

FD U-Net architecture was implemented for artifact removal in OA imaging. FD U-Net approach applies dense connectivity in both up-sampling and down-sampling paths. The fundamental difference between U-Net and FD U-Net lies in replacing the 3×3 convolution with a dense block. Note that in FD U-Net the feature map size is reduced in the encoder path (using max-pooling operation) and increased in the decoder path (using deconvolution). At each spatial step, the dense block has a growth rate of k_s, which increases the learnable features from the input to f_s. The user can define initial values of these two hyperparameters, and the general representation of the growth rate expressed as, $k_s = 2^{s-1} \times k_1$ and $f_s = 2^{s-1} \times f_1$. At each spatial levels the features extracted from the up-sampling operation and features extracted from the down-sampling operations are concatenated. This concatenation yields $2 \times f_s$ features, this cannot be processed by dense block. Hence at each spatial level 1×1 convolution layer was used to reduce the features by half in the decoder path. The details of FD U-Net architecture was shown in Fig. 2 and 3.

1.2 Y-Net and FD Y-Net architecture

Y-Net as opposed to other networks can take two inputs (data [sinogram] space and image space), so it has two encoder paths and one decoder path. Note that the spatial dimension of the data space and image space need not match, hence dimensionality mismatch (due to inconsistency of data and image space dimension) arises during concatenation in the encoder path. This issue was overcome by resizing the spatial dimension of each layer during encoding. The resizing operation was performed in the convolutional layers and the network learns the corresponding parameters during the training process. The Y-Net approach learns structural information about the input image and the results seem to enhance due to the usage of two encoder paths. The used Y-Net architecture was shown Fig. 4.

FD Y-Net architecture is implemented on the same lines as the FD U-Net architecture, the two convolution layers are replaced by a single dense block.

1.3 Deep residual U-Net architecture

Deep ResUnet has an encoder-decoder architecture used for semantic segmentation. Initially Deep ResUNet was used for road extraction from aerial images, later adopted by researchers for medical imaging applications. Deep ResUnet has the advantages of both the U-Net architecture and the Deep Residual Learning. Basic building blocks of this network is the residual block, which consists of convolution layers, activation layers, and batch normalization layers. Identity Mapping in the form of skip connection was used to connect the input and the output of the residual block. Note that identity mapping enables the flow of data in both forward and backward directions from one block to any other block. In Deep ResUnet, spatial dimension size was reduced by using a normal convolution layer with a stride of 2. The lower level extracted feature maps are concatenated with the corresponding higher-level extracted feature maps, thus this network has the ability to up-sample the spatial dimension size. The architecture detail was shown in Fig. 5.

1.4 GAN architecture

The Generative Adversarial Network (GAN) consists of two CNN networks namely a Generator and a Discriminator. Generator network takes fluence affected image as input and generates a fluence corrected image as the output and tries to fool the discriminator network. On the other hand, the discriminator network receives the input generated from the generator (fake image) and from the reference dataset (real image). These two images are used by the discriminator network to distinguish between the real and fake images. This can be achieved by a min-max optimization algorithm where the generator tries to minimize the joint loss function in contrast the discriminator wants to maximize this joint loss function, which is called adversarial training. The GAN network architecture for generator and discriminator networks were shown in Fig. 6.

Fig. 1- Fig. 7 shows the different DL model architectures which we implemented for our study. For GAN implementation we used Deep Residual U-Net as Generator network and it is shown in Fig. 5 and the Discriminator network was show in Fig. 6. Fig. 8 shows the line plot for different 2D slices, which gives more confirmation of the fluence correction by DL models

References

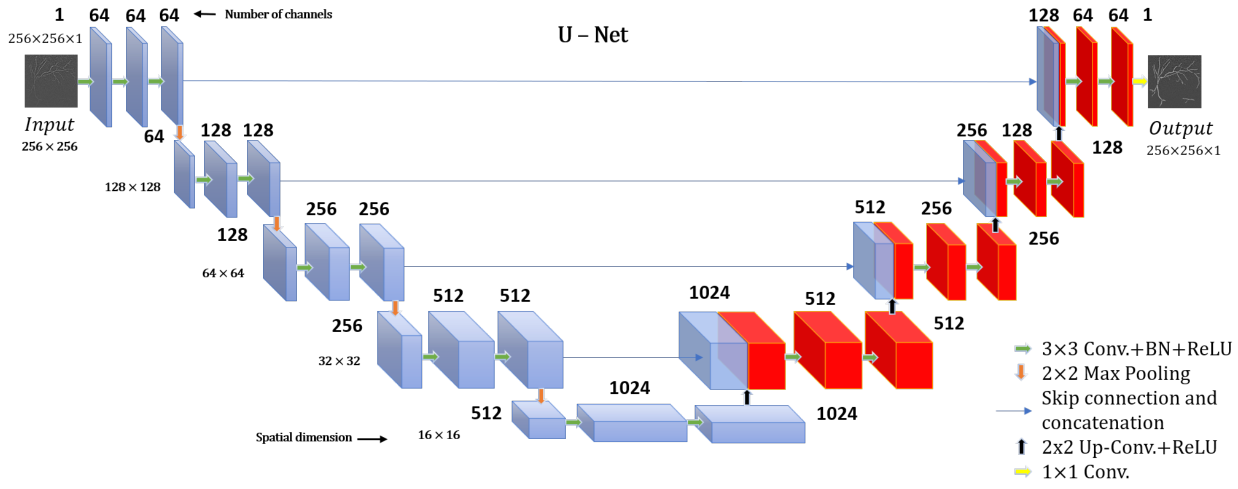

Fig 1 U-Net architecture

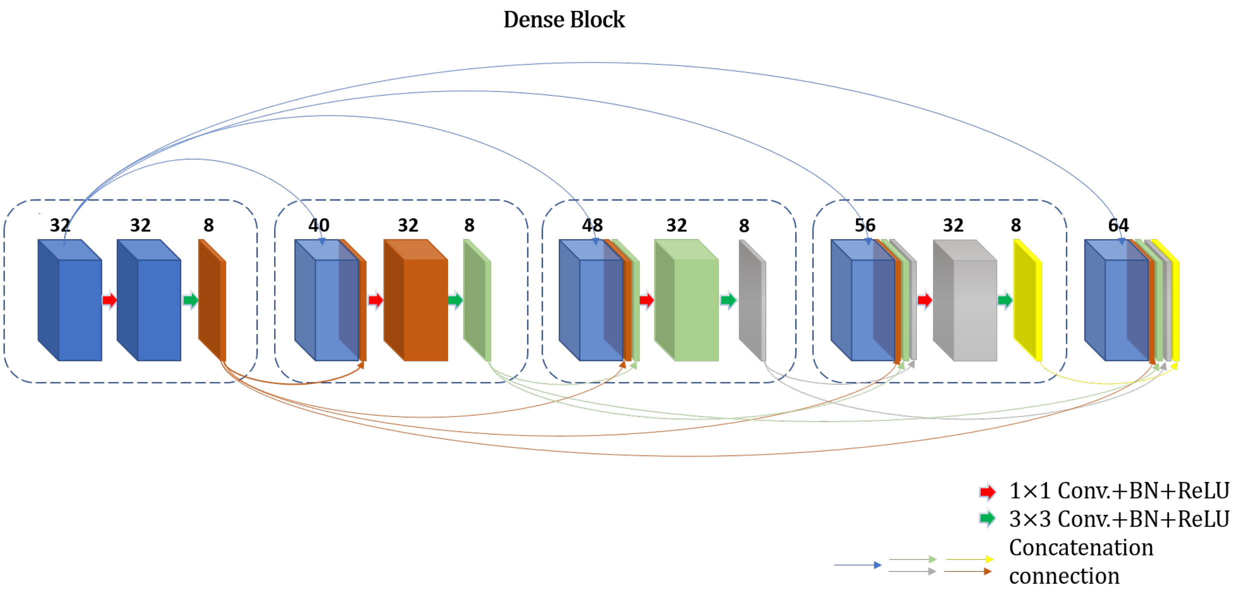

Fig 2 Dense block

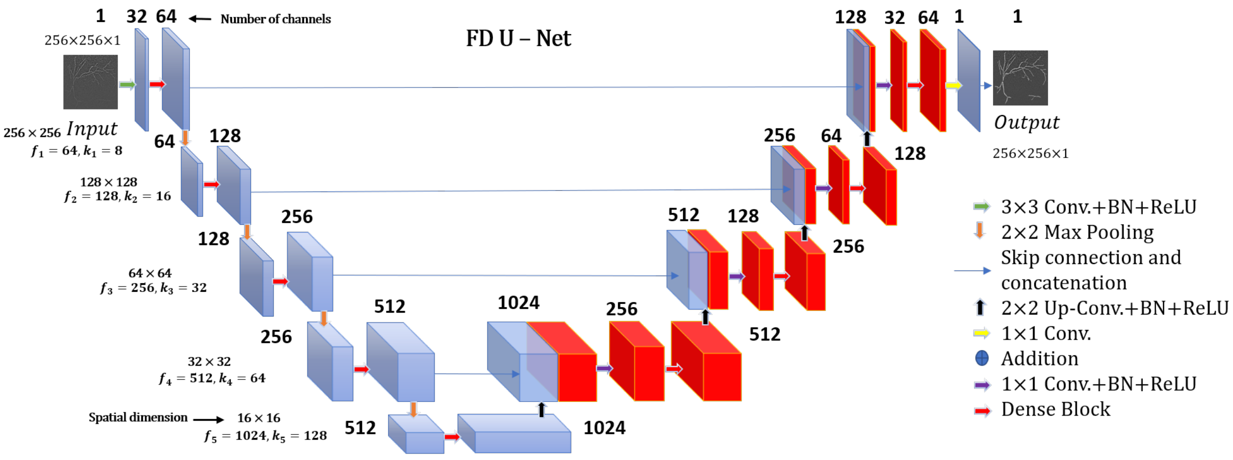

Fig 3 FD U-Net architecture

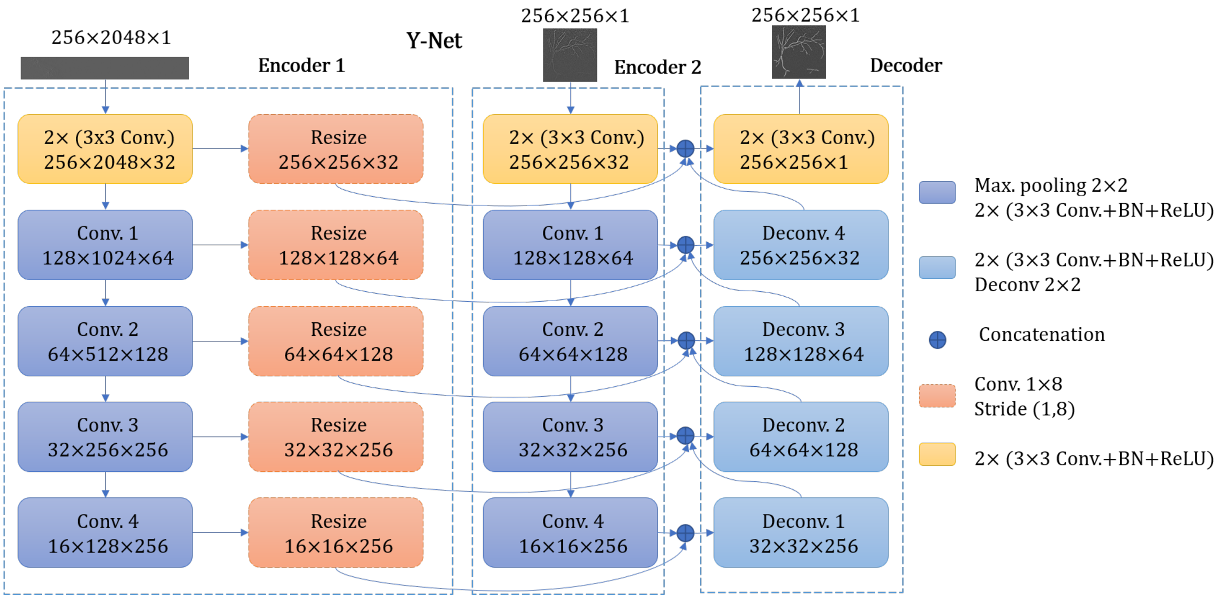

Fig 4 Y-Net architecture

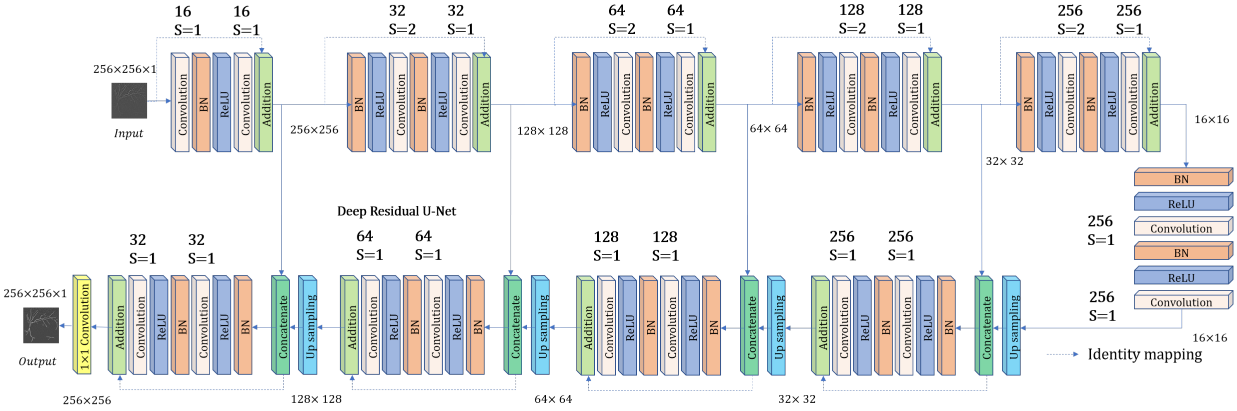

Fig 5 Deep Residual U-Net architecture

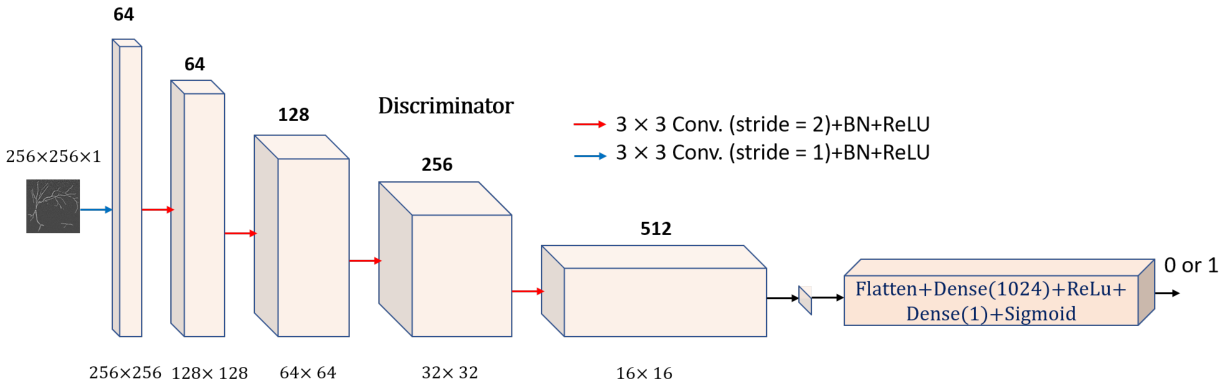

Fig 6 Discriminator

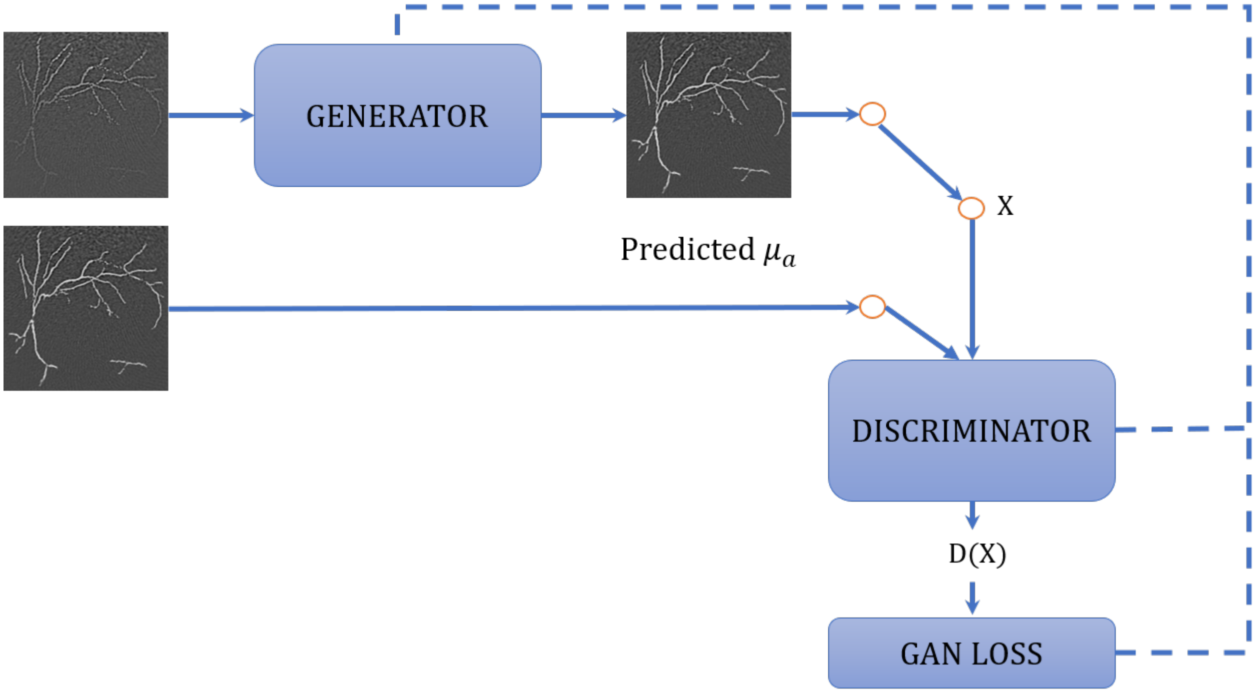

Fig 7 GAN

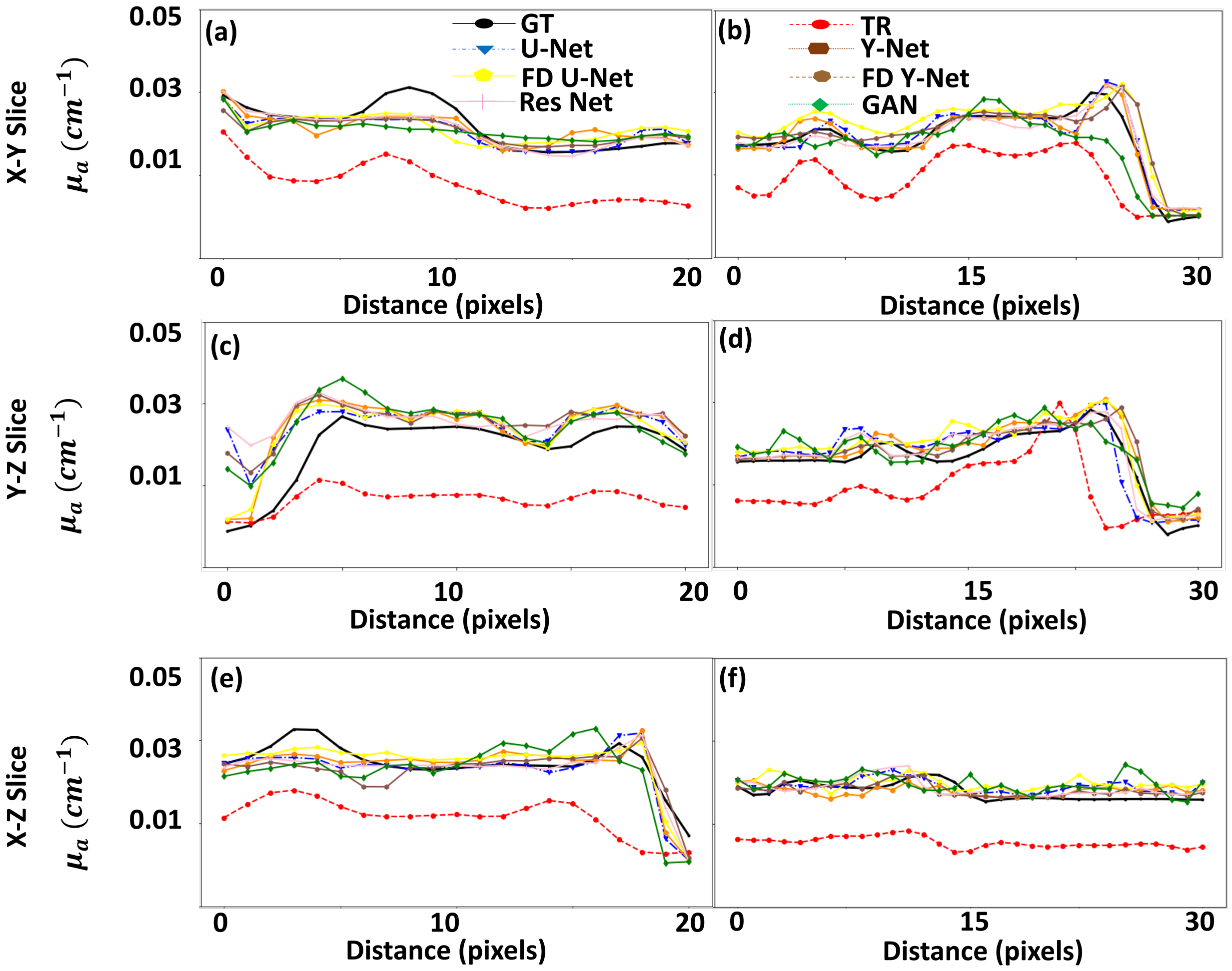

Fig 8 Intensity profiles along the Orange and Blue lines drawn on 3D PA reconstructed images.
